# Local recurrence after local excision of early rectal cancer: a meta‐analysis of completion TME, adjuvant (chemo)radiation, or no additional treatment

**DOI:** 10.1002/bjs.12040

**Published:** 2020-09-16

**Authors:** S. E. van Oostendorp, L. J. H. Smits, Y. Vroom, R. Detering, M. W. Heymans, L. M. G. Moons, P. J. Tanis, E. J. R. de Graaf, C. Cunningham, Q. Denost, M. Kusters, J. B. Tuynman

**Affiliations:** ^1^ Department of Surgery Amsterdam UMC, Vrije Universiteit Amsterdam, Cancer Centre Amsterdam Amsterdam the Netherlands; ^2^ Department of Surgery, Amsterdam UMC University of Amsterdam, Cancer Centre Amsterdam Amsterdam the Netherlands; ^3^ Department of Epidemiology and Biostatistics, Amsterdam UMC Vrije Universiteit Amsterdam Amsterdam the Netherlands; ^4^ Department of Gastroenterology University Medical Centre Utrecht Utrecht the Netherlands; ^5^ Department of Surgery, IJsselland Ziekenhuis, Capelle aan den Ijssel the Netherlands; ^6^ Department of Surgery Oxford University Hospitals Oxford UK; ^7^ Department of Surgery Centre Hospitalier Universitaire de Bordeaux Bordeaux France

## Abstract

**Background:**

The risks of local recurrence and treatment‐related morbidity need to be balanced after local excision of early rectal cancer. The aim of this meta‐analysis was to determine oncological outcomes after local excision of pT1–2 rectal cancer followed by no additional treatment (NAT), completion total mesorectal excision (cTME) or adjuvant (chemo)radiotherapy (aCRT).

**Methods:**

A systematic search was conducted in PubMed, Embase and the Cochrane Library. The primary outcome was local recurrence. Statistical analysis included calculation of the weighted average of proportions.

**Results:**

Some 73 studies comprising 4674 patients were included in the analysis. Sixty‐two evaluated NAT, 13 cTME and 28 aCRT. The local recurrence rate for NAT among low‐risk pT1 tumours was 6·7 (95 per cent c.i. 4·8 to 9·3) per cent. There were no local recurrences of low‐risk pT1 tumours after cTME or aCRT. The local recurrence rate for high‐risk pT1 tumours was 13·6 (8·0 to 22·0) per cent for local excision only, 4·1 (1·7 to 9·4) per cent for cTME and 3·9 (2·0 to 7·5) per cent for aCRT. Local recurrence rates for pT2 tumours were 28·9 (22·3 to 36·4) per cent with NAT, 4 (1 to 13) per cent after cTME and 14·7 (11·2 to 19·0) per cent after aCRT.

**Conclusion:**

There is a substantial risk of local recurrence in patients who receive no additional treatment after local excision, especially those with high‐risk pT1 and pT2 rectal cancer. The lowest recurrence risk is provided by cTME; aCRT has outcomes comparable to those of cTME for high‐risk pT1 tumours, but shows a higher risk for pT2 tumours.

## Graphical abstract

This meta‐analysis showed that patients who undergo no additional treatment after local excision of pT1–2 rectal cancer have a high risk of local recurrence, especially those with high‐risk pT1 and pT2 lesions. The risk of local recurrence after adjuvant (chemo)radiotherapy for high‐risk pT1 tumours seems to be similar to that after completion TME. For pT2 tumours, adjuvant (chemo)radiotherapy seems less effective than radical surgery.

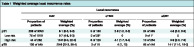

No additional therapy after local excision of pT1‐2 associated with high risk of local recurrence

## Abstract (es)

**Antecedentes:**

Tras una resección temprana de un cáncer de recto localizado, hay que considerar el equilibrio entre el riesgo de recidiva local y la morbilidad relacionada con el tratamiento. El objetivo de este metaanálisis era determinar los resultados oncológicos tras la resección de un cáncer de recto pT1‐T2 seguida de ningún tratamiento adicional (*no additional treatment*, NAT), escisión total del mesorrecto (*completion total mesorectal excision*, cTME) o quimiorradioterapia adyuvante (*adjuvant chemoradiotherapy*, aCRT).

**Methods:**

Se llevó a cabo una búsqueda sistemática en PubMed, Embase y biblioteca Cochrane. La variable principal de resultado era la recidiva local (*local recurrence*, LR). En el análisis estadístico se calcularon las medias ponderadas de proporciones.

**Resultados:**

Se incluyeron en el análisis 76 estudios con un total de 4.793 pacientes. NAT fue evaluada en 72 estudios, cTME en 13 y aCRT en 28. La tasa de LR para NAT en tumores pT1 de bajo riesgo era de 6,7% (i.c. del 95% 4,8‐9,3). No se observaron casos de LR en tumores pT1 de bajo riesgo tras cTME o aCRT. La tasa de LR para tumores pT1 de alto riesgo fue de 13,6% (i.c. del 95% 8,0‐22,0) para la resección local como único tratamiento, 4,1% (i.c. del 95% 1,7‐9,4) para cTME y 3,9% (i.c. del 95% 2,0‐7,5) para aCRT. La tasa de LR para tumores pT2 fue de 28,9% (i.c. del 95% 22,3‐36,4) para NAT, 4,3% (i.c. del 95% 1,4‐12,5) para cTME y 14,7% (i.c. del 95% 11,2‐19,0) para aCRT.

**Conclusión:**

Tras la resección local de cáncer pT1 de alto riesgo y pT2, existe un riesgo sustancial de recidiva local en ausencia de tratamiento adicional. La escisión total del mesorrecto se asocia con el menor riesgo de recidiva. La quimiorradioterapia adyuvante ofrece resultados similares a la escisión total del mesorrecto en tumores pT1 de alto riesgo, pero presenta un mayor riesgo en tumores pT2.

## Introduction

Screening programmes for bowel cancer have resulted in a substantial shift towards earlier stages of colorectal cancer^1^, ^2^. Apart from low‐risk pT1 tumours, the current standard treatment for rectal cancer is total mesorectal excision (TME) with or without neoadjuvant (chemo)radiotherapy depending on tumour stage^3^. This radical approach is associated with morbidity, long‐term functional impairment and consequently a decrease in quality of life^4^, ^5^. The increased incidence of early lesions, treatment‐related morbidity and the impact of treatment on quality of life create a clinical need for organ preservation, especially in patients with early rectal cancer^3^, ^6^.

Clinical staging by endoscopy, MRI and endoscopic ultrasonography (EUS) has low accuracy in distinguishing low‐risk T1 from high‐risk T1 or early T2 rectal cancers^7^, ^8^. Therefore, local excision as an initial diagnostic procedure is an attractive approach in early rectal cancer. This might turn out to be therapeutic in selected patients based on the histopathological results, and is associated with low morbidity and good functional outcomes^9^. Local excision is not considered oncologically safe for high‐risk pT1 tumours because of a higher risk of recurrence^3^, ^10^, ^11^. Despite the recommendations of guidelines, patients and physicians often refrain from completion TME (cTME) for high‐risk tumours^12^. Clinical data supporting this strategy are scarce and relatively high recurrence rates have been reported^11^, ^13^, ^14^, ^15^, ^16^.

A promising organ‐sparing alternative after local excision is adjuvant (chemo)radiotherapy (aCRT), which is being evaluated in trials^17^. Long‐term outcome data for all treatment options are essential in developing a valid clinical decision‐making algorithm for both patients and physicians.

The aim of this meta‐analysis was to provide an update on a previous meta‐analysis, and to evaluate the increasing amount of data for the three treatment strategies after local excision of pT1–2 rectal cancer: no additional treatment (NAT), cTME and aCRT^18^. Local recurrence rates, distant recurrence rates and both disease‐free (DFS) and overall survival (OS) rates were evaluated for these treatment strategies.

## Methods

### Search strategy

The study was performed according to the PRISMA guidelines^19^. Comprehensive searches regarding the treatment options were performed in the bibliographic databases of PubMed, Embase and the Cochrane Library for NAT (*Appendix* *S1*, supporting information) and for cTME and aCRT (*Appendix* *S2*, supporting information). In contrast to the previous meta‐analysis^18^, NAT was added as a treatment option and an additional subgroup analysis was performed for low‐ and high‐risk pT1 tumours. Literature searches were carried out on 26 August 2019 and contained all available records to the date of the search. Studies were reviewed for eligibility by two independent researchers and a third in the event of discrepancies.

Studies were considered eligible if pT1–2 rectal carcinomas were included, treated with local excision followed by either NAT, cTME or aCRT, and met the following inclusion criteria: local recurrence rates were reported, a minimum of ten patients were included, articles were published since 1990 in the English language, and median length of follow‐up was at least 36 months. Exclusion criteria were neoadjuvant treatment, distant metastasis at the time of local excision and studies that included patients with suspected nodal metastases on MRI. Studies that did not describe pT category, treatment modality or the distinction between colonic or rectal cancer were considered ineligible. Animal studies, studies with overlapping data, reviews and letters were excluded.

### Quality assessment

To assess the quality of the included studies, the Methodological Index for Non‐Randomized Studies (MINORS) instrument was used^20^. Each item was scored independently by two authors from 0 to 2 points: 0, not reported; 1, reported inadequately; and 2, reported adequately. In addition to the eight established elements, an item considering allocation bias was added to evaluate whether the treatment was chosen according to a protocol or surgeon's preference, or whether the rationale was not described.

### Outcome measures and statistical analysis

The primary outcome was local recurrence, defined as endoluminal recurrence or nodal recurrence in the pelvis. This included patients with isolated local recurrence as well as those with distant metastases. Secondary outcomes were distant metastasis, DFS and OS. Subgroup analyses were performed to differentiate outcomes by tumour category (pT1 *versus* pT2). A subgroup analysis for low‐ and high‐risk pT1 tumours was also included. Low‐ and high‐risk tumours were analysed separately if the presence of risk factors was described. High‐risk tumours were defined as lesions with at least one of the following histopathological risk factors: lymphovascular invasion, poor differentiation, deep submucosal invasion (sm3, Haggitt 4 or at least 1000 μm), tumour budding or positive resection margins (margin less than 1 mm or tumour in resection plane)^21^, ^22^. These factors had to be absent for tumours to be considered low risk. A weighted average of proportions was calculated using the generic inverse‐variance method and a random‐effects model. After natural log transformation of the individual proportions, the final results were back‐transformed.

**Fig. 1:**
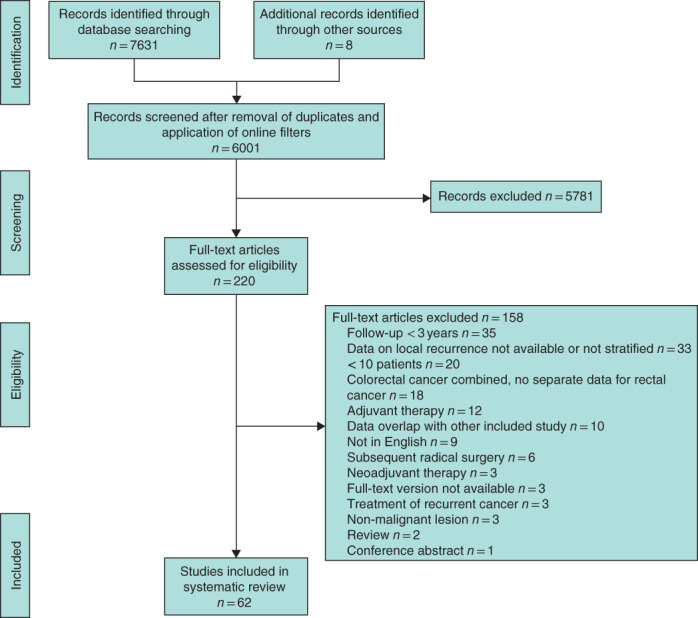
PRISMA flow chart showing selection of articles concerning local excision of early rectal cancer without additional treatment

Heterogeneity was assessed by means of the *I* ^2^ statistic; an *I* ^2^ value of 75–100 per cent represented considerable heterogeneity, so 75 per cent was used as the cut‐off value^23^. One pooled analysis of NAT in pT2 tumours showed statistically significant heterogeneity (*I* ^2^ = 55 per cent, *P* < 0·010) but was retained in the analyses. Owing to heterogeneous and scarce reporting of OS and DFS, no weighted averages were determined for these outcomes, but the range is presented. Survival rates were not incorporated if studies included patients with tumours other than pT1–2 lesions, without specified survival rates. DFS was defined as survival without local or distant recurrence.

## Results

### Included studies

**Fig. 2:**
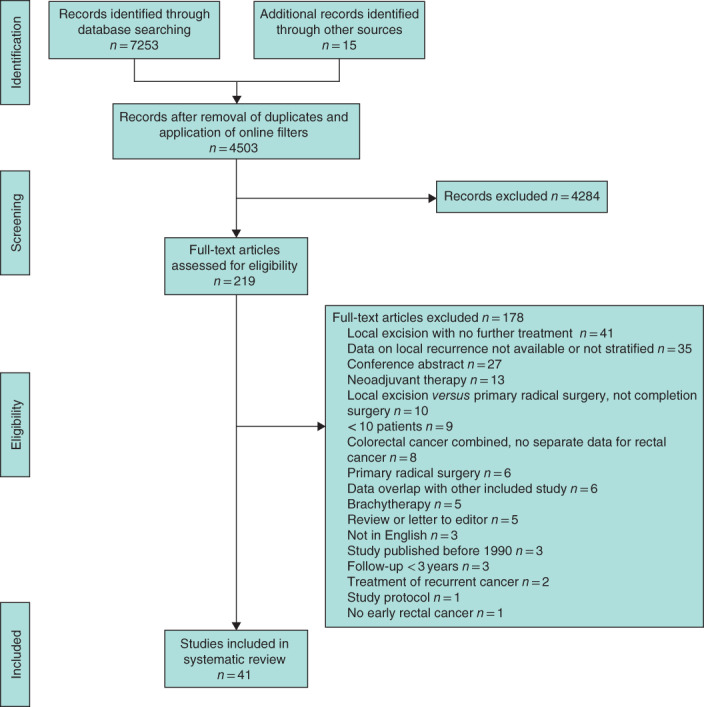
PRISMA flow chart showing selection of articles concerning adjuvant (chemo)radiotherapy or completion total mesorectal excision after local excision of early rectal cancer

A total of 73 cohort studies were included in this systematic review and meta‐analysis, compared with 19 in the previous meta‐analysis^18^ (*Figs* *1* and *2*). Sixty‐two publications^11^, ^13^, ^14^, ^15^, ^16^, ^24^, ^25^, ^26^, ^27^, ^28^, ^29^, ^30^, ^31^, ^32^, ^33^, ^34^, ^35^, ^36^, ^37^, ^38^, ^39^, ^40^, ^41^, ^42^, ^43^, ^44^, ^45^, ^46^, ^47^, ^48^, ^49^, ^50^, ^51^, ^52^, ^53^, ^54^, ^55^, ^56^, ^57^, ^58^, ^59^, ^60^, ^61^, ^62^, ^63^, ^64^, ^65^, ^66^, ^67^, ^68^, ^69^, ^70^, ^71^, ^72^, ^73^, ^74^, ^75^, ^76^, ^77^, ^78^, ^79^, ^80^ on local excision only were included, comprising 3050 patients with pT1 and 545 with pT2 disease (*Table S1*, supporting information). Thirteen studies^11^, ^33^, ^35^, ^44^, ^56^, ^59^, ^60^, ^61^, ^73^, ^78^, ^81^, ^82^, ^83^ reported outcomes of local excision followed by cTME, comprising 180 patients with pT1 and 70 with pT2 tumours (*Table S2*, supporting information). Finally, 28 studies^24^, ^28^, ^29^, ^32^, ^34^, ^35^, ^38^, ^41^, ^42^, ^48^, ^52^, ^54^, ^59^, ^60^, ^63^, ^64^, ^67^, ^72^, ^74^, ^75^, ^84^, ^85^, ^86^, ^87^, ^88^, ^89^, ^90^, ^91^ of aCRT were included, and contained 385 patients with pT1 and 444 with pT2 lesions (*Table S3*, supporting information). For the subgroup analysis of low‐risk pT1 tumours, a total of 29 studies described no additional treatment, one cTME and one aCRT. Among studies of high‐risk pT1 tumours, NAT was administered in 19, cTME in seven and aCRT in 12. Twenty‐nine of the 62 studies of local excision alone reported active surveillance during follow‐up. For aCRT, 14 of the 28 studies reported close follow‐up schemes. Ten of the 73 included studies (14 per cent) were prospective cohort studies. Of these, eight concerned NAT after local excision, one included patients who underwent cTME and three evaluated aCRT.

Different local excision techniques were used in the included studies. In the NAT group, 52 of the 62 studies evaluated surgical modalities of local excision, five investigated endoscopic local excisions, and five studied either both or did not describe the local excision technique. For cTME, nine of 13 studies evaluated surgical local excision, three investigated endoscopic local excision, and one included both surgical and endoscopic techniques. Among studies of aCRT, 24 of 28 evaluated surgical excision techniques, whereas four investigated both surgical and endoscopic techniques or did not describe the excision method used (*Tables S1–S3*, supporting information).

### Local recurrence

For patients with a pT1 tumour, the weighted local recurrence rate was 8·1 (95 per cent c.i. 6·6 to 9·9) per cent without additional treatment, 2·8 (1·2 to 6·5) for those who had cTME and 4·8 (2·3 to 9·8) among patients who received aCRT (*Table* *1*). For low‐risk pT1 tumours, NAT was associated with a local recurrence rate of 6·7 (4·8 to 9·3) per cent. No local recurrences were reported after cTME or aCRT in patients with low‐risk pT1 lesions, based on one study for each treatment strategy. Weighted local recurrence rates for high‐risk pT1 tumours were 13·6 (8·0 to 22·0), 4·1 (1·7 to 9·4) and 3·9 (2·0 to 7·5) per cent for NAT, cTME and aCRT respectively.

**Table 1:** Weighted average local recurrence rates

	Local recurrence					
	NAT		cTME		aCRT	
	Proportion of patients	Weighted average (%)	Proportion of patients	Weighted average (%)	Proportion of patients	Weighted average (%)
**pT1**	268 of 3050	8·1 (6·6, 9·9)	5 of 180	2·8 (1·2, 6·5)	24 of 385	4·8 (2·3, 9·8)
Low risk	75 of 1019	6·7 (4·8, 9·3)	0 of 28*	0	0 of 1*	0
High risk	44 of 282	13·6 (8·0, 22·0)	5 of 123	4·1 (1·7, 9·4)	10 of 254	3·9 (2·0, 7·5)
**pT2**	136 of 545	28·9 (22·3, 36·4)	3 of 70	4 (1, 13)	66 of 444	14·7 (11·2, 19·0)

Values in parentheses are 95 per cent confidence intervals.

* Results from a single study. NAT, no additional treatment; cTME, completion total mesorectal excision; aCRT, adjuvant (chemo)radiotherapy.

In patients with a pT2 tumour, NAT was associated with a local recurrence rate of 28·9 (22·3 to 36·4) per cent. This analysis showed significant heterogeneity (*I* ^2^ = 55 per cent; *P* < 0·010) (*Fig. S1*, supporting information). For pT2 tumours, the local recurrence rate was 4 (1 to 13) per cent after cTME and 14·7 (11·2 to 19·0) per cent following aCRT.

Patients with local recurrences were defined as patients with either local recurrence only, or those with both local and distant recurrence (*Table S4*, supporting information). Study‐specific local recurrence rates are reported in *Tables S5–S10* and *Figs S1–S6* (supporting information).

### Distant metastasis

The weighted distant metastasis rate in patients with pT1 tumours was 3·4 (95 per cent c.i. 2·5 to 4·6) per cent in those who had NAT, 4·9 per cent (2·4 to 9·4) per cent after cTME and 5·0 (3·0 to 8·3) per cent after aCRT (*Table* *2*; *Tables S5–S7* and *Figs S7–S9*, supporting information). Weighted average distant metastasis rates for pT2 tumours were 6·2 (2·8 to 13·0) per cent for NAT, 7 (3 to 18) per cent for cTME and 5·8 (2·7 to 11·9) per cent for aCRT.

**Table 2:** Weighted average distant recurrence rates

	Distant recurrence					
	NAT		cTME		aCRT	
	Proportion of patients	Weighted average (%)	Proportion of patients	Weighted average (%)	Proportion of patients	Weighted average (%)
**pT1**	101 of 2658	3·4 (2·5, 4·6)	8 of 165	4·9 (2·4, 9·4)	14 of 280	5·0 (3·0, 8·3)
Low risk	25 of 783	3·2 (2·2, 4·7)	1 of 28*	4	0 of 1*	0
High risk	20 of 233	7·2 (3·6, 13·9)	6 of 108	5·6 (2·5, 11·8)	8 of 208	3·9 (1·9, 7·5)
**pT2**	28 of 398	6·2 (2·8, 13·0)	4 of 55	7 (3, 18)	17 of 254	5·8 (2·7, 11·9)

Values in parentheses are 95 per cent confidence intervals.

* Results from a single study. NAT, no additional treatment; cTME, completion total mesorectal excision; aCRT, adjuvant (chemo)radiotherapy.

### Survival

Five‐year DFS for local excision without additional treatment of pT1 tumours was reported in eight publications (range 67–97 per cent), with two of these studies reporting a 5‐year DFS rate above 85 per cent (*Table* *3*; *Tables S5–S7*, supporting information). Only one study reported a 5‐year DFS rate after cTME, which was 81 per cent. For aCRT of pT1 tumours, the 5‐year DFS rate was reported in six studies (range 59–100 per cent), of which five reported a rate of more than 85 per cent. For pT2 tumours, the 5‐year DFS with NAT was reported in three publications as 65, 81 and 93 per cent. One study reported a 3‐year DFS of 100 per cent after cTME. Four studies reported 5‐year DFS rates for treatment of pT2 tumours with aCRT, which ranged from 58 to 78 per cent.

**Table 3:** Five‐year overall and disease‐free survival rates

	NAT			cTME		aCRT		
	*n*	Survival rate (%)	Reported survival > 85%	*n*	Survival rate (%)	*n*	Survival rate (%)	Reported survival > 85%
**pT1**								
Disease‐free survival	8	67–97	2 of 8	1	81	6	59–100	5 of 6
Overall survival	15	65–100	5 of 15	1	92	6	63–98	3 of 6
**pT2**								
Disease‐free survival	3	65–93	1 of 3	1	100*	4	58–78	0 of 4
Overall survival	7	30–95	2 of 7		n.r.	5	58–93	2 of 5

* Three‐year disease‐free survival. NAT, no additional treatment; cTME, completion total mesorectal excision, aCRT, adjuvant (chemo)radiotherapy; *n*, number of studies reporting this value; n.r., not reported.

The 5‐year OS rate after NAT of pT1 tumours was reported in 15 studies (range 65–100 per cent) and exceeded 85 per cent in one‐third of these (*Table* *3*; *Tables S5–S7*, supporting information). After cTME, the 5‐year OS rate was 92 per cent in one study. Five‐year OS following aCRT was reported in six studies (range 63–98 per cent), half of which showed a rate of over 85 per cent. For pT2 tumours, the 5‐year OS rate in patients without additional treatment was reported in seven publications (range 30–95 per cent), and two of these studies described an OS rate of more than 85 per cent. Five studies reported 5‐year OS after aCRT for pT2 tumours (range 58–93 per cent); the rate exceeded 85 per cent in two of these studies.

### Study quality assessment

Study assessment according to the MINORS checklist revealed that almost 90 per cent of the included studies were carried out retrospectively (*Tables S11–S12*, supporting information).

## Discussion

This study showed that patients who undergo NAT after local excision of pT1–2 rectal cancer have a high risk of local recurrence, especially those with high‐risk pT1 and pT2 lesions. For high‐risk pT1 tumours, the risk of local recurrence after aCRT seems similar to that for cTME. For pT2 tumours, aCRT seems less effective than radical surgery. The study findings could be used to support both patients and clinicians in decision‐making.

A recent review^92^ of local recurrence rates for pT1 colorectal tumours excised endoscopically without additional treatment identified an overall recurrence rate of 9 per cent for rectal cancer. The present data showed that high‐risk pT1 is associated with a relatively high risk of recurrence of 13·6 per cent after local excision alone, which is consistent with previous findings^46^, ^53^. An older large cohort study^10^ reported an even higher rate of 19 per cent local recurrence for pT1 tumours. The relatively high local recurrence rate of 29 per cent for locally excised pT2 tumours in the same study corresponds to the present findings. Other studies^93^, ^94^ have confirmed high local recurrence rates for pT2 cancer, and cTME is recommended.

Data on aCRT after local excision of early rectal cancer are scarce. Most series are hampered by a lack of standardized histopathological evaluation distinguishing low‐ from high‐risk pT1 lesions. One of the largest cohort studies^86^ of 83 patients reported a 3·6 per cent rate of local recurrence. In a review by Cutting and colleagues^95^, local recurrence rates were comparable to those of the present analysis: 5·8 per cent for pT1 and 13·8 per cent for pT2 tumours. An earlier meta‐analysis by the present study group^18^, which did not incorporate patients without additional treatment, reported a higher local recurrence rate of 10 per cent for pT1 tumours, and a similar rate of 15 per cent for pT2 tumours. One of the largest series of tumours resected endoscopically followed by cTME, reported by Tamaru and co‐workers^73^, included 56 pT1 tumours and showed a local recurrence rate of 4 per cent. Borschitz *et al*.^11^ described the largest number of cTMEs after transanal endoscopic microsurgery, and reported a local recurrence rate of 5 per cent for high‐risk pT1 tumours and 10 per cent for pT2 tumours, which are higher than the pooled rates reported here.

The occurrence of distant metastases was comparable for the three treatment strategies, with weighted average rates ranging between 3·4 and 5·0 per cent for pT1 lesions, and from 5·8 to 7 per cent for pT2 lesions. This is lower than rates reported elsewhere. In a study^96^ of locally excised pT2–3 rectal cancers, distant metastases were observed in 16 per cent at 3 years of follow‐up of patients who underwent NAT or transanal endoscopic microsurgery followed by cTME. In the previous review^18^, the weighted average distant recurrence rate was 9 per cent in patients treated with aCRT or cTME.

The type of treatment is not expected to influence the occurrence of distant metastasis. However, aspects such as tumour biology and the development of local recurrence may influence the risk of distant metastasis. These hypotheses cannot be confirmed based on the present review, but are in line with the findings of other studies^97^, ^98^.

The intensity of surveillance of patients who received NAT varied among the studies. About half of the studies reported endoscopic, MRI and/or EUS surveillance every 3–4 months during the first 2 or 3 years after local excision. A large proportion of the studies (31 of 73) did not report specific follow‐up schemes. Active surveillance of both local and distant recurrences is crucial in an organ‐preserving strategy for high‐risk tumours. Unfortunately, the type (endoluminal or nodal) and stage of local recurrences were not reported in the majority of the included studies. Few studies have reported eligibility and outcomes of salvage treatment in the event of local recurrence after local excision^99^, ^100^, ^101^. Based on this limited evidence, the proportion of patients deemed eligible for salvage surgery ranges from 73 to 93 per cent^13^, ^100^, ^101^, ^102^, ^103^. Salvage surgery is associated with more extensive procedures and low rates of sphincter preservation. Weiser and colleagues^104^ described a cohort of 50 patients who underwent salvage surgery, of whom 55 per cent required extended pelvic resection. In three studies^100^, ^101^, ^105^ of salvage treatment, the sphincter could not be preserved in approximately two‐thirds of the patients who underwent salvage surgery. Moreover, survival rates are low in patients eligible for curative salvage surgery. Several studies^100^, ^104^, ^106^ have reported 5‐year OS rates of around 50 per cent after salvage treatment. Limited data are available on cancer‐specific survival following salvage treatment. Doornebosch *et al*.^13^ reported a 3‐year cancer‐specific survival rate of 58 per cent, and Vaid and colleagues^103^ a 5‐year cancer‐specific survival rate of 53 per cent. A systematic review^99^ also reported a disappointing 5‐year OS rate of 50 per cent after salvage surgery, presumably owing to the increased incidence of distant metastasis. Conceivably, with adequate follow‐up, local recurrences might be detected at an early stage. If clear resection margins were achieved, the 5‐year OS rate was estimated at 59 per cent by Weiser and colleagues^104^, compared with 0 per cent for incomplete resections.

Although the present data seem more robust than those in previous reports, there remains a lack of high‐quality data and appropriate reporting of long‐term outcomes of local treatment for early rectal cancer, which emphasizes the need for clinical trials^18^. The advantages and disadvantages (morbidity, function and oncological outcomes) of the three treatment options should be considered for each patient individually. The increase in risk of recurrence that is acceptable in order to preserve the rectum is unclear, and may differ between patients and physicians. Eventually, the decision regarding rectum‐preserving treatment depends on both patient preferences and tumour characteristics, and should be based on shared decision‐making.

An alternative strategy to accomplish organ preservation is the use of neoadjuvant chemoradiotherapy, which has been shown to downsize tumours and even lead to complete remission in over 50 per cent of patients ^107^, ^108^. However, patients without complete remission require TME surgery. This implies that neoadjuvant chemoradiotherapy led to overtreatment of patients with non‐responding or partially responding tumours, and likely resulted in increased morbidity. More importantly, as clinical staging by imaging has been shown to lack accuracy, this treatment strategy also incorporates patients with low‐risk tumours, who could have been treated with local excision only^7^, ^8^. For this reason, a strategy comprising local excision of small lesions without signs of risk factors on preoperative imaging seems more attractive. Based on histopathological risk factors, additional treatment can be tailored to the individual patient.

The present meta‐analysis is based on extensive data from 73 studies, compared with 19 in the previous meta‐analysis^18^. Besides the newly added third treatment strategy, NAT, for which 62 studies were included, the number of studies evaluating cTME and aCRT was doubled to 13 and 28 respectively. Yet, the present analysis was limited by the heterogeneity of the included studies and selection bias in allocated treatment. Variation in follow‐up protocols, duration of follow‐up, sample size and type of adjuvant treatment was observed. In some studies, patients underwent radiotherapy without concurrent chemotherapy. Patients unfit for surgery and those who refused additional treatment were often allocated to NAT, presumably leading to selection bias. Owing to the variability in follow‐up, local recurrence rates were not correlated with follow‐up duration or protocols. Despite these methodological differences, it was decided to perform a pooled analysis. Quality assessment according to the MINORS checklist revealed that nearly 90 per cent of the included studies were retrospective. Many studies did not describe the histopathological inclusion criteria in detail, and definitions of histopathological risk factors varied; for example, some studies reported a margin of less 1 mm as a risk factor, whereas others defined a positive resection margin by the presence of carcinoma in the resection plane. Moreover, deep submucosal infiltration was determined to be a histopathological risk factor. However, more recent evidence shows that deep submucosal invasion alone is not a strong risk factor for lymph node metastases in multivariable analysis^109^. Nevertheless, subgroup analysis for low‐ and high‐risk pT1 tumours was undertaken because it provides important information for clinical decision‐making, and reporting only overall local recurrence rates would have led to additional bias. The data on pT2 tumours are heterogeneous, and probably include a proportion of patients with nodal disease as a result of under‐reporting of inclusion criteria and suspected nodal involvement on preoperative imaging. Furthermore, patients with unidentified nodal disease might have been included in studies of NAT and aCRT, whereas such patients were excluded from analyses of cTME. These issues may have influenced the outcomes. Survival data were not reported sufficiently to allow pooling, and might have been influenced by the selection of patients for each treatment strategy. For these reasons, only ranges could be described and no conclusions could be drawn based on the available data. A potential confounder is the method of local excision. The majority of included studies evaluated surgical local excision techniques. Further research is needed to explore differences in outcomes within and between surgical and endoscopic techniques for local excision^110^, ^111^. In addition, the location of local recurrence (endoluminal, mesorectal or lymph node involvement) was generally not reported, but is of value in decision‐making for salvage treatment. Despite these limitations, an attempt was made to minimize heterogeneity by applying strict inclusion criteria and reporting data for the included subgroups only.

## Supporting information


**Appendix** **S1 Search details of local excision without additional treatment**
Appendix **S2 Search details of adjuvant (chemo)radiation and completion TME following local excision**
**Table S1 Characteristics of studies on local excision without additional treatment for early rectal cancer**
**Table S2 Characteristics of studies on local excision followed by completion TME for early rectal cancer**
**Table S3 Characteristics of studies on local excision followed by adjuvant (chemo)radiation for early rectal cancer**
Table S4 Proportions of local recurrence, either local recurrence only or local recurrence and distant metastases.
**Table S5 Outcome data of local excision without additional treatment**
**Table S6 Outcome data of local excision followed by completion total mesorectal excision**
**Table S7 Outcome data of local excision followed by adjuvant (chemo)radiation**
**Table S8 Outcome data of local excision without additional treatment, subgroup analysis low‐ and high‐risk pT1.**
**Table S9 Outcome data of local excision followed by completion total mesorectal excision, subgroup analysis low‐ and high‐risk pT1**
**Table S10 Outcome data of local excision followed by adjuvant (chemo)radiotherapy, subgroup analysis low‐ and high‐risk pT1**
**Table S11 Quality assessment of studies on no additional treatment after local excision**
**Table S12 Quality assessment of studies on completion total mesorectal excision and adjuvant (chemo)radiation following local excision**
**Figure S1 Forest plots of overall local recurrence of local without additional treatment in patients with a) pT1 and b) pT2 tumours. An inverse‐variance random‐effects model. Proportions with 95 per cent confidence intervals**
**Figure S2 Forest plots of overall local recurrence of local excision followed by completion total mesorectal excision in patients with a) pT1 b) pT2 tumours. An inverse‐variance random‐effects model. Proportions with 95 per cent confidence intervals**
**Figure S3 Forest plots of overall local recurrence of local excision followed by adjuvant (chemo)radiotherapy in patients with a) pT1 b) pT2 tumours. An inverse‐variance random‐effects model. Proportions with 95 per cent confidence intervals**
**Figure S4 Forest plots of overall local recurrence of local excision without additional treatment, subgroup analysis low‐ and high‐risk T1 tumours a) low‐risk pT1 and b) high‐risk pT1 tumours. An inverse‐variance random‐effects model. Proportions with 95 per cent confidence intervals**
**Figure S5 Forest plots of overall local recurrence of local excision followed by completion total mesorectal excision, subgroup analysis high‐risk pT1 tumours. An inverse‐variance random‐effects model. Proportions with 95 per cent confidence intervals**
**Figure S6 Forest plots of overall local recurrence of local excision followed by adjuvant (chemo)radiotherapy, subgroup analysis high‐risk pT1 tumours. An inverse‐variance random‐effects model. Proportions with 95 per cent confidence intervals**
**Figure S7 Forest plots of overall distant recurrence of local excision without additional treatment in patients with a) pT1, b) pT2 tumours. An inverse‐variance random‐effects model. Proportions with 95 per cent confidence intervals.**
**Figure S8 Forest plots of overall distant recurrence of local excision followed by completion total mesorectal excision in patients with a) pT1 and b) pT2 tumours. An inverse‐variance random‐effects model. Proportions with 95 per cent confidence intervals**
**Figure S9 Forest plots of overall distant recurrence of local excision followed by adjuvant (chemo)radiotherapy in patients with a) pT1 and b) pT2 tumours. An inverse‐variance random‐effects model. Proportions with 95 per cent confidence intervals**
