# Supplementary material for: Local recurrence after local excision of early rectal cancer: a meta‐analysis of completion TME, adjuvant (chemo)radiation, or no additional treatment
Source: Br J Surg. 2020 Sep 16;107(13):1719–30. doi: 10.1002/bjs.12040 (PMC7692925; doi:10.1002/bjs.12040)

**BJS12040**

**Local recurrence rates associated with completion total mesorectal excision, adjuvant (chemo)radiation or no additional treatment after local excision of pT1–2 rectal cancer: a meta-analysis**

S. E. van Oostendorp, L. J. H. Smits, Y. Vroom, R. Detering, M. W. Heymans, L. M. G. Moons, P. J. Tanis, E. J. R. de Graaf, C. Cunningham, Q. Denost, M. Kusters and J. B. Tuynman

**Appendix S1 Search details of local excision without additional treatment**

### Pubmed

| Query | Items found |
| --- | --- |
| ((“Watchful Waiting"[Mesh] OR "Follow-Up Studies"[Mesh] OR watchful waiting*[tiab] OR conservative*[tiab] OR noninvasive[tiab] OR non-invasive[tiab] OR nonsurgical[tiab] OR non-surgical[tiab] OR observation[tiab] OR watch-and-wait[tiab] OR wait and see[tiab] OR surveillance[tiab] OR follow-up[tiab] OR follow up[tiab]) AND  ("Natural Orifice Endoscopic Surgery"[Mesh] OR "Microsurgery"[Mesh] OR "Minimally Invasive Surgical Procedures"[Mesh] OR microsurger*[tiab] OR microsurgical*[tiab] OR TEM[tiab] OR TAMIS[tiab] OR EMR[tiab] OR ESD[tiab] OR endoscopic mucosal resection[tiab] OR transanal*[tiab] OR local excision[tiab] OR transanal excision*[tiab] OR full thickness excision*[tiab] OR rectoscop*[tiab] OR endoscop*[tiab] OR locally excised[tiab] OR teo[tiab]) AND  ("Rectal Neoplasms"[Mesh] OR rectal neoplasm*[tiab] OR rectal cancer*[tiab] OR rectal carcinoma*[tiab] OR rectal tumor*[tiab] OR rectal tumor*[tiab] OR rectal malignan*[tiab] OR rectum neoplasm*[tiab] OR rectum cancer*[tiab] OR rectum carcinoma*[tiab] OR rectum tumor*[tiab] OR rectum tumor*[tiab] OR rectum malignan*[tiab] OR early rectal cancer*[tiab] OR T1 rectal cancer*[tiab] OR T2 rectal cancer*[tiab])) | 2762 |

### Embase

| Query | Items found |
| --- | --- |
| (('watchful waiting'/exp OR 'follow up'/exp OR ‘watchful waiting*’:ab,ti,kw OR conservative*:ab,ti,kw OR noninvasive:ab,ti,kw OR ‘non-invasive’:ab,ti,kw OR nonsurgical:ab,ti,kw OR ‘non-surgical’:ab,ti,kw OR observation:ab,ti,kw OR ‘watch-and-wait’:ab,ti,kw OR ‘wait and see’:ab,ti,kw OR surveillance:ab,ti,kw OR ‘follow-up’:ab,ti,kw OR ‘follow up’:ab,ti,kw) AND  ('natural orifice transluminal endoscopic surgery'/exp OR 'microsurgery'/exp OR 'minimally invasive surgery'/exp OR 'transanal endoscopic surgery'/exp OR microsurger*:ab,ti,kw OR microsurgical*:ab,ti,kw OR TEM:ab,ti,kw OR TAMIS:ab,ti,kw OR EMR:ab,ti,kw OR ESD:ab,ti,kw OR ‘endoscopic mucosal resection’:ab,ti,kw OR transanal*:ab,ti,kw OR ‘local excision’:ab,ti,kw OR ‘transanal excision*’:ab,ti,kw OR ‘full thickness excision*’:ab,ti,kw OR rectoscop*:ab,ti,kw OR endoscop*:ab,ti,kw OR ‘locally excised’:ab,ti,kw OR teo:ab,ti,kw) AND  ('rectum tumor'/de OR 'colorectal tumor'/exp OR 'rectum cancer'/exp OR ((rectal OR rectum) AND (neoplasm* OR cancer* OR carcinoma* OR tumor* OR tumor* OR malignan* OR neoplasm* OR cancer* OR carcinoma* OR tumor* OR tumor* OR malignan*)):ab,ti,kw) NOT  (NOT ('conference abstract'/it OR 'conference review'/it OR 'editorial'/it OR 'letter'/it OR 'note'/it OR 'short survey'/it))) | 4463 |

### Cochrane

| Query | Items found |
| --- | --- |
| ((watchful waiting* or conservative* or noninvasive or "non-invasive" or nonsurgical or "non-surgical" or observation or "watch-and-wait" or "wait and see" or surveillance or "follow-up" or "follow up":ti,ab,kw (Word variations have been searched)) AND  (microsurger* or microsurgical* or TEM or TAMIS or EMR or ESD or "endoscopic mucosal resection" or transanal* or "local excision" or "transanal excision*" or "full thickness excision*" or rectoscop* or endoscop* or "locally excised" or teo:ti,ab,kw (Word variations have been searched)) AND  (((rectal or rectum) and (neoplasm* or cancer* or carcinoma* or tumor* or tumor* or malignan* or neoplasm* or cancer* or carcinoma* or tumor* or tumor* or malignan*)):ti,ab,kw (Word variations have been searched))) | 406 |

## Results

| Pubmed | 2762 |
| --- | --- |
| Embase.com | 4463 |
| The Cochrane Library (Wiley) | 198 |
| Total | 7631 |

**Appendix S2** **Search details of adjuvant (chemo)radiation and completion TME following local excision**

### Pubmed

| Query | Items found |
| --- | --- |
| (("Chemotherapy, Adjuvant"[Mesh] OR "Chemoradiotherapy, Adjuvant"[MeSH Terms] OR "Radiotherapy, Adjuvant"[MeSH Terms] OR adjuvant[tiab] OR completion surgery[tiab] OR total mesorectal excision[tiab] OR TME[tiab] OR TME surgery[tiab] OR radical surgery) AND  ("Microsurgery"[Mesh] OR "Minimally Invasive Surgical Procedures"[Mesh] OR transanal endoscopic microsurgery[tiab] OR microsurgery[tiab] OR microsurgical*[tiab] OR transanal minimally invasive surgery[tiab] OR TEM[tiab] OR TAMIS[tiab] OR EMR[tiab] OR ESD[tiab] OR endoscopic mucosal resection[tiab] OR local excision[tiab] OR transanal*[tiab] OR rectoscop*[tiab] OR endoscop*[tiab] OR limited[tiab]) AND  ("Rectal Neoplasms"[Mesh] OR rectal neoplasm*[tiab] OR rectal cancer*[tiab] OR rectal carcinoma*[tiab] OR rectal tumor*[tiab] OR rectal tumor*[tiab] OR rectal malignan*[tiab] OR rectum neoplasm*[tiab] OR rectum cancer*[tiab] OR rectum carcinoma*[tiab] OR rectum tumor*[tiab] OR rectum tumor*[tiab] OR rectum malignan*[tiab])) | 2600 |

### Embase

| Query | Items found |
| --- | --- |
| (('adjuvant chemotherapy'/exp OR 'adjuvant chemoradiotherapy'/exp OR adjuvant OR 'colorectal surgery'/exp OR 'tme surgery':ti,ab OR 'total mesorectal excision'/exp OR 'radical surgery':ti,ab OR 'completion surgery':ti,ab) AND  ('microsurgery'/exp OR 'minimally invasive surgery'/exp OR 'transanal endoscopic microsurgery':ti,ab OR microsurgery:ti,ab OR microsurgical*:ti,ab OR 'transanal minimally invasive surgery':ti,ab OR tem:ti,ab OR tamis:ti,ab OR emr:ti,ab OR esd:ti,ab OR 'endoscopic mucosal resection':ti,ab OR 'local excision':ti,ab OR transanal*:ti,ab OR rectoscop*:ti,ab OR endoscop*:ti,ab OR limited:ti,ab) AND  ('rectum tumor'/de OR 'colorectal tumor'/exp OR 'rectum cancer'/exp OR 'rectal neoplasm*':ab,ti OR 'rectal cancer*':ab,ti OR 'rectal carcinoma*':ab,ti OR 'rectal tumor*':ab,ti OR 'rectal tumor*':ab,ti OR 'rectal malignan*':ab,ti OR 'rectum neoplasm*':ab,ti OR 'rectum cancer*':ab,ti OR 'rectum carcinoma*':ab,ti OR 'rectum tumor*':ab,ti OR 'rectum tumor*':ab,ti OR 'rectum malignan*':ab,ti)) | 4486 |

### Cochrane

| Query | Items found |
| --- | --- |
| (((MeSH descriptor: [Rectal Neoplasms] explode all trees) OR  (rectal cancer* or rectal carcinoma*OR rectal tumor* or rectal tumor* or rectal malignan* or rectum neoplasm* or rectum cancer* or rectum carcinoma* or rectum tumor* or rectum tumor* or rectum malignan*:ti,ab,kw (Word variations have been searched))) AND  ((MeSH descriptor: [Minimally Invasive Surgical Procedures] explode all trees) OR  (MeSH descriptor: [Microsurgery] explode all trees) OR  (transanal endoscopic microsurgery or microsurgical* or transanal minimally invasive surgery or TEM or TAMIS or EMR or ESD or endoscopic mucosal resection or local excision or transanal* or rectoscop* or endoscop* or limited or TME or total mesorectal excision or completion surgery or radical surgery or colorectal surgery:ti,ab,kw (Word variations have been searched))) AND  ((MeSH descriptor: [Chemotherapy, Adjuvant] explode all trees) OR  (MeSH descriptor: [Chemotherapy, Adjuvant] explode all trees) OR  (MeSH descriptor: [Chemoradiotherapy, Adjuvant] explode all trees))) | 167 |

## Results

| Pubmed | 2600 |
| --- | --- |
| Embase.com | 4486 |
| The Cochrane Library (Wiley) | 167 |
| Total | 7253 |

**Table S1 Characteristics of studies on local excision without additional treatment for early rectal cancer**

| *Reference* | *Year of publication* | *Size of cohort* | *pT category* | *Local excision technique* | *Length of follow-up period (months)* | *Reason for follow-up without additional therapy* |
| --- | --- | --- | --- | --- | --- | --- |
| *Kwakye* | 2019 | 114 | T1 (n=114) | TAE/TEM/TAMIS | 64 (median) | n.r. |
| *Kouyama* | 2018 | 50 | T1 (n=50) | EMR/ESD/ polypectomy | 41.5 (mean) | Patients were either unfit or refused radical surgery. |
| *Jones* | 2018 | 60 | T1 (n=60) | TEM | 39.6 (median) | Patients were referred for radical surgery if the TEM specimen showed adverse histological features. Some patients were unfit for radical surgery or refused (n=10). |
| *Balyasnikova* | 2017 | 14 | T1 (n=14) | TEM/TAE | 49.0 (median) | Patients were referred for radical surgery if the TEM specimen showed adverse histological features. If radical surgery was declined patients could opt for adjuvant CHRT or intense follow-up (n=8). |
| *O'Neill* | 2017 | 55 | T1 (n=50) T2 (n=5) | TEM | 55.2 (median) | Current policy: T1N0 without adverse histologic features requires TEM alone |
| *Tamaru* | 2017 | 29 | T1 (n=29) | EMR/ESD/polypectomy | 86.6 (mean) | Patients were unfit for radical surgery. |
| *Stornes* | 2016 | 94 | T1 (n=66) T2 (n=28) | TEM | 60.0 (mean) | Patients were selected for TEM surgery based on patient and tumour characteristics as given by national guidelines. |
| *Restivo* | 2016 | 37 | T1 (n=32) T2 (n=5) | TEM | 52.5 (median) | The total study sample consisted of low risk pT1 tumours or patients with high risk tumours that refused further treatment. |
| *Junginger* | 2016 | 110 | T1 (n=110) | TEM | 103.2 (median) | Patients were selected for TEM surgery based on patient and tumour characteristics as given by national guidelines. |
| *Turza* | 2016 | 28 | T1 (n=28) | TAE | 64.0 (median) | n.r. |
| *Amann* | 2015 | 12 | T2 (n=12) | TEM | 47.3 (mean) | n.r. |
| *Lee, S* | 2015 | 35 | T1 (n=35) | EMR/ESD/TAE | 70.5 (mean) | Patients were either unfit or refused radical surgery. |
| *Bacic* | 2014 | 17 | T1 (n=15) T2 (n=2) | TEM | 42.0 (median) | n.r. |
| *Elmessiry* | 2014 | 64 | T1 (n=38) T2 (n=26) | TAE/TEM | 35.0 (median) | n.r. |
| *Guerrieri* | 2014 | 110 | T1 (n=110) | TEM | 82.0 (median) | Patients with pT1 tumours were referred for local excision. All pT2-3 tumours received radiotherapy with or without chemotherapy. |
| *Sun* | 2014 | 56 | T1 (n=16) T2 (n=40) | TAE | 120.0 (mean) | n.r. |
| *Ikematsu* | 2013 | 53 | T1 (n=53) | ER | 60.5 (median) | Some patients refused the subsequent radical resection that was recommended. |
| *Im* | 2013 | 53 | T1 (n=53) | LE | 60.0 (median) | n.r. |
| *Luglio* | 2013 | 18 | T1 (n=16) T2 (n=2) | LE | 60.0 (mean) | Patients with pT1 tumours were referred for local excision. Local excision was also performed in elderly patients that were unfit or refused major surgery (n=2) |
| *Amann* | 2012 | 39 | T1 (n=39) | TEM | 34.4 (mean) | n.r. |
| *Morino* | 2011 | 62 | T1 (n=48) T2 (n=14) | TEM | 39.5 (median) | Patients were either unfit or refused radical surgery. |
| *Oka* | 2011 | 83 | T1 (n=83) | ER | 38.7 (mean) | n.r. |
| *Ramirez* | 2011 | 53 | T1 (n=53) | TEM | 71.0 (mean) | The total study consisted of pT1 tumours deemed feasible for TEM. |
| *Doornebosch* | 2010 | 88 | T1 (n=88) | TEM | 30.0 (median) | The total study consisted of pT1 tumours deemed feasible for TEM. |
| *Peng* | 2010 | 58 | T1 (n=58) | TAE | 72.0 (median) | The total study sample consisted of low risk tumours or patients with high risk tumours that refused further treatment. |
| *Tsai* | 2010 | 63 | T1 (n=51) T2 (n= 12) | TEM | 49.5 (mean) | n.r. |
| *Allaix* | 2009 | 37 | T1 (n=35) T2 (n=12) | TEM | 60.0 (mean) | All pT2 tumours were referred for radical resection. Some patients refused (n=7) radical surgery or were unfit (n=11). |
| *Choi* | 2009 | 20 | T1 (n=20) | ER | 33.0 (median) | n.r. |
| *Huh* | 2009 | 33 | T1 (n=22) T2 (n=11) | TEM | 66.0 (median) | n.r. |
| *Lebedyev* | 2009 | 37 | T1 (n=37) | TEM/TAE | 57.0 (mean) | n.r. |
| *Borschitz* | 2008 | 136 | T1 (n=117) T2 (n=19) | TEM | 60.0 (median) | High-risk pT1 tumours, inadequate resections and pT2 tumours were referred for radical resection. Some patients refused radical resection (n=43). |
| *Duek* | 2008 | 4 | T2 (n=4) | TEM | 36 (median) | Patients were either unfit or refused further treatment. |
| *Greenberg* | 2008 | 59 | T1 (n=59) | TAE/transsphincteric/transrectal | 85.2 (median) | The total study consisted of pT1 tumours deemed feasible for TEM. |
| *Serra-Aracil* | 2008 | 25 | T1 (n=16) T2 (n=9) | TEM | 59.0 (median) | n.r. |
| *Lee, WY* | 2007 | 6 | T1 (n=6) | TEM | 62 (median) | Patients were either unfit or refused further treatment. |
| *Min* | 2007 | 37 | T1 (n=36) T2 (n=1) | LE | 84.9 (median) | The total study sample consisted of low risk tumours or patients with high risk tumours that refused further treatment. |
| *Ptok* | 2007 | 99 | T1 (n=99) | TEM/TAE | 44.0 (median) | The total study consisted of pT1 tumours deemed feasible for TEM. |
| *You* | 2007 | 765 | T1 (n=601)  T2 (n=164) | Local excision ICD-9-CM 48.3, 48.35, 48.36 | T1 75.6 T2 68.4 (median) | n.r. |
| *Zacharakis* | 2007 | 21 | T1 (n=14) T2 (n=7) | TEM | 37.0 (median) | Patients were either unfit or refused radical surgery. |
| *Floyd* | 2006 | 52 | T1 (n=52) | TEM | 34.1 (mean) | n.r. |
| *Ganai* | 2006 | 19 | T1 (n=19) | TEM | 43.0 (mean) | n.r. |
| *Maslekar* | 2006 | 46 | T1 (n=27) T2 (n=19) | TEM | 40.0 (median) | The total study consisted of pT1 tumours deemed feasible for TEM and patients with pT2 tumours that were either unfit or refused radical surgery. |
| *Endreseth* | 2005 | 35 | T1 (n=35) | TAE | 24-97 (range) | n.r. |
| *Gopaul* | 2004 | 42 | T1 (n=28) T2 (n=14) | LE | 37.0 (median) | n.r. |
| *Nascimbeni* | 2004 | 70 | T1 (n=70) | TAE | 110.4 (mean) | Patients were either unfit or refused radical surgery. |
| *Araki* | 2003 | 22 | T1 (n=22) | V-TEM | 61.0 (mean) | pT1 tumours with high-risk factors were referred for radical resection or adjuvant therapy. Some patients refused (n=4). |
| *Gonzalez* | 2003 | 15 | T1 (n=15) | TAE | 40.7 (median) | The total study consisted of pT1 tumours deemed feasible for TEM. |
| *Lee, W* | 2003 | 74 | T1 (n=52) T2 (n=22) | TEM | 31.0 (mean) | pT2 tumours were referred for radical surgery. Some patients refused (n=16) radical surgery or were high-risk patients (n=6). |
| *Nakagoe* | 2002 | 17 | T1 (n=15) T2 (n=2) | Gasless VTEM | 52.3 (median) | pT1 tumours with high-risk factors or pT2 tumours were referred for radical resection. Patients refused (n=1) or were high risk patients (n=2). |
| *Paty* | 2002 | 94 | T1 (n=67) T2 (n=27) | TAE/Kraske/transsphincteric | 80.4 (median) | n.r. |
| *Wykypiel* | 2002 | 15 | T1 (n=15) | TAE | 57.1 (median) | n.r. |
| *Budhoo* | 2000 | 22 | T1 (n=10) T2 (n=12) | TAE | Minimum of 5 years or until death | The total study sample was treated with local excision only. |
| *Garcia-Aguilar* | 2000 | 82 | T1 (n=55) T2 (n=27) | TAE | 54.0 (mean | n.r. |
| *Lamont* | 2000 | 23 | T1 (n=17) T2 (n=6) | TAE | 33.0 (median) | T2: 6 refused further therapy |
| *Mellgren* | 2000 | 108 | T1 (n=69) T2 (n=39) | TAE | 52.8 (mean) | n.r. |
| *Russell* | 2000 | 14 | T1 (n=14) | Transanal/transsacral/transcoccygeal | 73.5 (median) | The total study consisted of pT1 tumours deemed feasible for TEM. |
| *Chakravarti* | 1999 | 52 | T1 (n=44)  T2 (n=8) | York-Mason/Kraske | 51 (median) | n.r. |
| *Heintz* | 1998 | 58 | T1 (n=58) | TEM | LR 52 (mean)  HR 42.8 (mean) | The total study sample consisted of low risk pT1 tumours or patients with high risk tumours that refused or were unfit for further treatment. |
| *Taylor* | 1998 | 23 | T1 (n=15) T2 (n= 8) | TAE | 52.0 (median) | Patients were either unfit or refused radical surgery. |
| *Bleday* | 1997 | 21 | T1 (n=21) | TAE/transsphincteric/transcoccygeal | 39.5 (median) | The total study consisted of pT1 tumours deemed feasible for TEM. |
| *Winde* | 1997 | 25 | T1 (n=25) | TEM | 40.9 (mean) | n.r. |
| *Coco* | 1995 | 22 | T1 (n=22) | TAE | 52 (median) | The total study sample consisted of low risk pT1 tumours, patients with high risk tumours were referred for radical resection. |

LE: local excision, ER: endoscopic resection, TAE: transanal excision, TEM: transanal endoscopic microsurgery, VTEM: video transanal endoscopic microsurgery, TAR: transanal resection, EMR: endoscopic mucosal resection, ESD: endoscopic submucosal dissection.

**Table S2 Characteristics of studies on local excision followed by completion TME for early rectal cancer**

| *Reference* | *Year of Publication* | *Size of cohort* | *pT category : n* | *local excision technique* | *Radical surgery* | *Length of follow-up (months)* |
| --- | --- | --- | --- | --- | --- | --- |
| *Antonelli* | 2018 | 13 | T1 (n=13) | Polypectomy/ESD/EMR | TME | 30 (median) |
| *Ortenzi* | 2018 | 30 | T1 (n=15)  T2 (n=15) | TEM | TME: APR | 108 (mean) |
| *Tamaru* | 2017 | 56 | T1 (n=56) | EMR/ESD/ polypectomy | TME | 104 (mean) |
| *Morino* | 2011 | 5 | T2 (n=5) | TEM | TME | 39.5 (median) |
| *Choi* | 2009 | 13 | T1 (n=13) | ER | TME | 33 (median) |
| *Borschitz* | 2008 | 39 | T1 (n=19)  T2 (n=20) | TEM | TME: LAR/AR/APR | 61 (median) |
| *Duek* | 2008 | 5 | T2 (n=5) | TEM | TME | 58 (median) |
| *Min* | 2007 | 7 | T2 (n=7) | TEM | TME: APR | 84.9 (median) |
| *Lee, WY* | 2007 | 9 | T1 (n=3)  T2 (n=6) | TEM | TME: LAR/APR | 62 (median) |
| *Hahnloser* | 2005 | 37 | T1 (n=28)  T2 (n= 9) | TAE/polypectomy | TME: LAR/APR | 120 (median) |
| *Nakagoe* | 2004 | 11 | T1 (n=8)  T2 (n=3) | Gasless VTEM | TME: LAR/APR | 86.5 (median) |
| *Wykypiel* | 2002 | 3 | T1 (n=2)  T2 (n=1) | TAE | TME | 57.1 (median) |
| *Heintz* | 1998 | 22 | T1 (n=22) | TEM | TME | 42.8 (mean) |

TAE: transanal excision, TEM: transanal endoscopic microsurgery, VTEM: video endoscopic microsurgery, TME: transanal mesorectal excision, APR: abdominal perineal resection, AR: anterior resection, LAR: low anterior resection, ER: endoscopic resection.

**Table S3 Characteristics of studies on local excision followed by adjuvant (chemo)radiation for early rectal cancer**

| *Reference* | *Year of publication* | *Size of cohort* | *pT category* | *local excision technique* | *Adjuvant therapy* | *Length of follow-up (months)* |
| --- | --- | --- | --- | --- | --- | --- |
| *Jones* | 2018 | 26 | T1 (n=8)  T2 (n=18) | TEM | 45/25 Gy + capecitbine or 25/5 Gy | 38.4 (median) |
| *Suzuki* | 2018 | 50 | T1 (n=50) | Transanal full thickness | 40-45/20-25 Gy + EFT or S1 | 71 (median) |
| *Balyasnikova* | 2017 | 18 | T1 (n=11) T2 (n=7) | TEM/TAR | 45/25 Gy + capecitabine / 9 / 5.4 Gy | 49.0 (median) |
| *O’Neill* | 2017 | 21 | T1 (n=4) T2 (n=17) | TEM | CRT: n.r. | 55.2 (median) |
| *Sasaki* | 2017 | 57 | T1 (n=53) T2 (n=4) | TAE | 45 Gy / 25 fractions of 1.8 Gy + 5-FU | 87.6 (median) |
| *Jeong* | 2016 | 83 | T1 (n=68) T2 (n=15) | TAE/EMR/ESD | 45.0 Gy in 1.8 Gy fractions / 5 weeks + 5.4 Gy + 5-FU + LV | 61.0 (median) |
| *Rackley* | 2016 | 88 | T1 (n=46) T2 (n=42) | TEM/TAE | 3-4 field 40.0-50.4 Gy | 74.0 (median) |
| *Lee, S* | 2015 | 31 | T1 (n=31) | EMR/ESD/TAE | 49.6 ± 3.6 Gy + 5-FU | 78.7 (mean) |
| *Bacic* | 2014 | 4 | T2 (n=4) | TEM | CRT: details n.r. | 47 (median) |
| *Sun* | 2014 | 49 | T1 (n=8)  T2 (n=41) | TAE | 15-50 Gy / 45 Gy / 10-60 Gy / 21 - 67 Gy | 120.0 (mean) |
| *Ramirez* | 2011 | 28 | T1 (n=6)  T2 (n=22) | TEM | 50.4/28 Gy | 71.0 (mean) |
| *Morino* | 2011 | 19 | T2 (n=19) | TEM | CRT: details n.r | 39.5 (median) |
| *Tsai* | 2010 | 5 | T2 (n=5) | TEM | CRT: details n.r. | 42 (mean) |
| *Allaix* | 2009 | 19 | T1 (n=3)  T2 (n=16) | TEM | CRT: details n.r. | 60 (mean) |
| *Greenberg* | 2008 | 51 | T2 (n=51) | TAE/transsphincteric/transrectal | 5-FU + 50.4/28 Gy | 85.2 (mean) |
| *Duek* | 2008 | 12 | T2 (n=12) | TEM | RT: details n.r. | 58.0 (median) |
| *Min* | 2007 | 19 | T1 (n=11)  T2 (n=8) | TEM | RT: 45/25+5⋅4Gy /  CRT: 5-FU+45/25+5⋅4Gy | 84.9 (median) |
| *Ganai* | 2006 | 1 | T1 (n=1) | TEM | CRT: 5-FU,RT not described | 43 (mean) |
| *Stipa* | 2006 | 6 | T1 (n=2)  T2 (n=4) | TEM | CRT: details n.r. | 78.0 (median) |
| *Gopaul* | 2004 | 15 | T1 (n=4)  T2 (n=11) | LE | RT: 45/25 Gy | 37.0 (median) |
| *Paty* | 2002 | 31 | T1 (n=7)  T2 (n=24) | TAE/ Kraske/transsphincteric | 4500-5400 cGy | 80.4 (mean) |
| *Benson* | 2001 | 60 | T1 (n=24)  T2 (n=36) | Transsacral | median 50 Gy (50/20 Gy), range 38.25-60 Gy | 48 (median) |
| *Lamont* | 2000 | 20 | T1 (n=10)  T2 (n=10) | TEM | CRT: 5-FU+45/25 or 46/22 Gy | 33.0 (median) |
| *Chakravarti* | 1999 | 47 | T1 (n=13)  T2 (n=33) | York-mason, Kraske | mean 53.6 Gy, 45/25 Gy + boost, 5-FU since 1986 | 51 (median) |
| *Wagman* | 1999 | 31 | T1 (n=6)  T2 (n=25) | LE | RT: 45/25+3⋅6–10⋅8Gy  CRT: 5-FU+RT | 41.0 (median) |
| *Taylor* | 1998 | 21 | T1 (n=12)  T2 (n=9) | TAE | 45–50/25 Gy | 52.0 (median) |
| *Valentini* | 1996 | 21 | T1 (n=9)  T2 (n=12) | TAE | 1.7Gy/day until 17 Gy (whole pelvis), followed by 2.0 Gy/day for 14 Gy (true pelvis), followed by 1.7 Gy/day until 13.6Gy | 54 (median) |
| *Coco* | 1995 | 15 | T2 (n=15) | TAE | 44⋅6/25 Gy | 52.0 (median) |

LE: local excision, TAE: transanal excision, TEM: transanal endoscopic microsurgery, TAR: transanal resection, EMR: endoscopic mucosal resection, ESD: endoscopic submucosal dissection, RT: radiotherapy, CRT: chemoradiotherapy, 5-FU: 5-fluoruracil, n.r.: not reported.

Table S4 Proportions of local recurrence, either local recurrence only or local recurrence and distant metastases.

|  | **NAT events (%)** | **cTME events (%)** | **aCRT events (%)** |
| --- | --- | --- | --- |
| **pT1** | 268 | 5 | 24 |
| LR | 252 (94.0) | 3 (60.0) | 18 (75.0) |
| LR + DR | 16 (6.0) | 2 (40.0) | 6 (25.0) |
| **pT1 low-risk** | 75 | 0 * | 0 * |
| LR | 68 (90.7) | 0 | 0 |
| LR + DR | 7 (9.3) | 0 | 0 |
| **pT1 high-risk** | 44 | 5 | 10 |
| LR | 41 (93.2) | 2 (40.0) | 5 (50.0) |
| LR + DR | 3 (6.8) | 3 (60.0) | 5 (50.0) |
| **pT2** | 136 | 3 | 66 |
| LR | 131 (96.3) | 3 (100.0) | 61 (92.4) |
| LR + DR | 5 (3.7) | 0 (0.0) | 5 (7.6) |

LR: local recurrence, DR: distant metastases, NAT: no additional treatment, cTME: completion total mesorectal excision, aCRT: adjuvant (chemo)radiotherapy. *One study

**Table S5 Outcome data of local excision without additional treatment**

| *Reference* | *Size of cohort* | *pT category* | Overall recurrence | Overall local recurrence | | Overall distant recurrence | | DFS % | OS % |
| --- | --- | --- | --- | --- | --- | --- | --- | --- | --- |
| *Kwakye* | 114 | T1 (n=114) | T1: 14 of 112 | | T1: 13 of 114 | | T1: 3 of 114 | n.r. | n.r. |
| *Kouyama* | 50 | T1 (n=50) | T1: 3 of 50 | | T1: 1 of 50 | | T1: 2 of 50 | n.r. | n.r. |
| *Jones* | 60 | T1 (n=60) | T1: 5 of 60 | | T1: 5 of 60 | | T1: 1 of 60 | T1: 3-yr 92.8 T2: 5-yr 92.8 | n.r. |
| *Balyasnikova* | 14 | T1 (n=14) | T1: 3 of 14 | | T1: 2 of 14 | | T1: 1 of 14 | n.r. | T1: 4-yr 100 |
| *O'Neill* | 55 | T1 (n=50) T2 (n=5) | T1: 4 of 50 T2: 0 of 5 | | T1: 2 of 50 T2: 0 of 5 | | T1: 2 of 50 T2: 0 of 5 | T1: 3-yr 94.3 | T1: 3-yr 81.8 |
| *Tamaru* | 29 | T1 (n=29) | T1: 1 of 29 | | T1: 1 of 29 | | T1: 0 of 29 | n.r. | n.r. |
| *Stornes* | 110 | T1 (n=110) | T1: 18 of 110 | | T1: 16 of 110 | | T1: 5 of 82 | n.r. | n.r. |
| *Restivo* | 37 | T1 (n=32) T2 (n=5) | T1: 1 of 32  T2: 4 of 5 | | T1: 1 of 32  T2: 4 of 5 | | n.r. | n.r. | n.r. |
| *Junginger* | 94 | T1 (n=66) T2 (n=28) | T1: 12 of 66 T2: 3 of 28 | | T1: 10 of 66 T2: 3 of 28 | | T1: 2 of 66 T2: 0 of 28 | n.r. | T1: 5-yr 65.3 T2: 5-yr 42.1 |
| *Turza* | 28 | T1 (n=28) | T1: 1 of 28 | | T1: 1 of 28 | | T1: 0 of 28 | n.r. | T1: 96.4 |
| *Amann* | 12 | T2 (n=12) | T2: 1 of 12 | | T2 : 1 of 12 | | n.r. | n.r. | n.r. |
| *Lee, S* | 35 | T1 (n=35) | T1: 4 of 35 | | T1: 2 of 35 | | T1: 2 of 35 | T1: 5-year 97.0 | n.r. |
| *Bacic* | 17 | T1 (n=15) T2 (n=2) | T1: 0 of 15 T2: 2 of 2 | | T1: 0 of 15 T2: 2 of 2 | | T1: 0 of 15 T2: 0 of 2 | n.r. | n.r. |
| *Elmessiry* | 64 | T1 (n=38) T2 (n=26) | T1: 8 of 38 T2: 13 of 26 | | T1: 7 of 38 T2: 11 of 26 | | T1: 1 of 38  T2: 2 of 26 | T1: 3-yr 84.2 T2: 3-yr 61.5 | T1: 3-yr 84.2 T2: 3-yr 61.5 |
| *Guerrieri* | 110 | T1 (n=110) | T1: 0 of 110 | | T1: 0 of 110 | | T1: 0 of 110 | n.r. | n.r. |
| *Sun* | 56 | T1 (n=16) T2 (n=40) | T1: 1 of 16 T2: 6 of 40 | | T1: 1 of 16 T2: 6 of 40 | | n.r. | n.r. | T1: 5-yr 75  T1: 10-yr 38 T2: 5-yr 30  T2: 10-yr 10 |
| *Ikematsu* | 53 | T1 (n=53) | T1: 7 of 53 | | T1: 4 of 53 | | T1: 3 of 53 | LR T1: 5-yr 90.0 HR T1: 5-yr 77.7 | LR T1: 5-yr 88.9 HR T1: 5-yr 96.2 |
| *Im* | 53 | T1 (n=53) | T1: 9 of 53 | | n.r. | | n.r. | n.r. | n.r. |
| *Luglio* | 18 | T1 (n=16) T2 (n=2) | T1: 1 of 16 T2: 2 of 2 | | T1 0 of 16 T2 2 of 2 | | T1 1 of 16 T2 2 of 2 | n.r. | n.r. |
| *Amann* | 39 | T1 (n=39) | T1: 4 of 39 | | 4 of 39 | | 1 of 39 | T1: 97.6 | n.r. |
| *Morino* | 62 | T1 (n=48) T2 (n=14) | T1: 5 of 48 T2: 5 of 14 | | T1: 5 of 48 T2: 5 of 14 | | T1: 0 of 48 T2: 0 of 14 | T1: 5-yr 85.9 T2: n.r. | T1: 5-yr 100 T2: n.r. |
| *Oka* | 83 | T1 (n=83) | T1: 8 of 83 | | T1: 2 of 83 | | T1: 6 of 88 | n.r. | n.r. |
| *Ramirez* | 53 | T1 (n=53) | T1: 4 of 53 | | T1: 3 of 53 | | T1: 1 of 53 | n.r. | n.r. |
| *Doornebosch* | 88 | T1 (n=88) | T1: 18 of 88 | | T1: 11 of 88 | | T1:7 of 88 | n.r. | n.r. |
| *Peng* | 58 | T1 (n=58) | T1: 6 of 58 | | T1: 6 of 58 | | n.r. | T1: 10-yrs: 85.5 | T1: 10-yrs: 85.4 |
| *Tsai* | 63 | T1 (n=51) T2 (n= 12) | T1: 9 of 51 T2: 3 of 12 | | T1: 5 of 51 T2: 3 of 12 | | T1: 4 of 51 T2: 0 of 12 | n.r. | n.r. |
| *Allaix* | 37 | T1 (n=35) T2 (n=12) | T1: 0 of 35 T2: 4 of 12 | | n.r. | | n.r. | n.r. | n.r. |
| *Choi* | 20 | T1 (n=20) | T1: 0 of 20 | | T1: 0 of 20 | | T1: 0 of 20 | n.r. | n.r. |
| *Huh* | 33 | T1 (n=22) T2 (n=11) | T1: 6 of 22 T2: 4 of 11 | | T1: 5 of 22 T2: 3 of 11 | | T1: 1 of 22 T2: 3 of 11 | T1 5-yr 71 T2 5-yr 64.8 | T1: 5-yr 93.3 T2: 5-yr 64.9 |
| *Lebedyev* | 37 | T1 (n=37) | T1: 2 of 37 | | T1: 2 of 37 | | T1:0 of 37 | T1 5 yr: 91 | T1: 5 yr: 81 |
| *Borschitz* | 136 | T1 (n=117) T2 (n=19) | T1: 21 of 117 T2: 13 of 19 | | T1: 15 of 117 T2: 7 of 19 | | T1: 6 of 117 T2: 6 of 19 | n.r. | n.r. |
| *Duek* | 4 | T2 (n=4) | T2 : 2 of 4 | | T2: 2 of 4 | | T2: 1 of 4 | n.r. | n.r. |
| *Greenberg* | 59 | T1 (n=59) | T1: 7 of 59 | | T1: 5 of 59 | | T1: 3 of 59 | T1: 10-yr: 75 | T1: 10-yr: 84 |
| *Serra-Aracil* | 25 | T1 (n=16) T2 (n=9) | T1: 1 of 16 T2: 3 of 9 | | T1: 1 of 16 T2: 2 of 9 | | T1: 0 of 16 T2: 1 of 9 | T1: 92.3 T2: 77.8 | T1: 100 T2: 82 |
| *Lee, WY* | 6 | T1 (n=6) | T1 : 1 of 6 | | T1: 1 of 6 | | T1: 1 of 6 | n.r. | n.r. |
| *Min* | 37 | T1 (n=36) T2 (n=1) | T1: 4 of 36 T2: 0 of 1 | | T1: 4 of 36 T2: 0 of 1 | | T1: 1 of 36 T2: 0 of 1 | n.r. | n.r. |
| *Ptok* | 99 | T1 (n=99) | T1: 9 of 99 | | T1: 5 of 99 | | T1: 4 of 99 | T1 5-yr 91.4 | T1 5-yr 83.6 |
| *You* | 765 | T1 (n=601)  T2 (n=164) | T1: 66 of 601  T2: 28 of 164 | | T1: 46 of 601  T2: 20 of 164 | | T1: 20 of 601  T2: 8 of 164 | n.r. | T1 5-yr: 77.4  T2: 5-yr: 67.6 |
| *Zacharakis* | 21 | T1 (n=14) T2 (n=7) | T1: 1 of 14 T2: 3 of 7 | | T1: 1 of 14 T2: 3 of 7 | | n.r. | n.r. | n.r. |
| *Floyd* | 52 | T1 (n=52) | T1: 3 of 52 | | T1: 3 of 52 | | T1: 0 of 52 | n.r. | n.r. |
| *Ganai* | 19 | T1 (n=19) | T1: 2 of 19 | | T1: 2 of 19 | | T1: 0 of 19 | T1: 5 yrs: 89 | T1: 5 yrs: 73 |
| *Maslekar* | 46 | T1 (n=27) T2 (n=19) | T1: 0 of 27 T2: 4 of 19 | | T1: 0 of 27 T2: 4 of 19 | | T1: 0 of 27 T2: 0 of 19 | n.r. | n.r. |
| *Endreseth* | 35 | T1 (n=35) | T1 4 of 35 | | T1 4 of 35 | | T1: 0 of 35 | n.r. | T1: 5-yr 70 |
| *Gopaul* | 42 | T1 (n=28) T2 (n=14) | T1: 3 of 28 T2: 5 of 14 | | T1: 3 of 28 T2: 5 of 14 | | n.r. | n.r. | n.r. |
| *Nascimbeni* | 70 | T1 (n=70) | T1: 17 of 70 | | T1: 6 of 70 | | T1: 11 of 70 | T1: 5-yr 66.6 T1: 10-yr 39.6 | T1: 5-yr 72.4 T1: 10-yr 44.3 |
| *Araki* | 22 | T1 (n=22) | T1: 0 of 22 | | T1: 0 of 22 | | T1: 0 of 22 | n.r. | n.r. |
| *Gonzalez* | 15 | T1 (n=15) | T1: 0 of 15 | | T1: 0 of 15 | | T1: 0 of 15 | n.r. | n.r. |
| *Lee, W* | 74 | T1 (n=52) T2 (n=22) | T1: 2 of 52 T2: 4 of 22 | | T1: 2 of 52 T2: 4 of 22 | | n.r. | T1: 5-yr 95.9 T2: 5-yr 80.5 | T1: 5-yr 100 T2: 5-yr 94.7 |
| *Nakagoe* | 17 | T1 (n=15) T2 (n=2) | T1: 0 of 15  T2: 0 of 2 | | T1: 0 of 15  T2: 0 of 2 | | T1: 0 of 15  T2: 0 of 2 | n.r. | n.r. |
| *Paty* | 94 | T1 (n=67) T2 (n=27) | T1: 14 of 67 T2: 9 of 27 | | T1: 10 of 67 T2: 7 of 27 | | T1: 4 of 67 T2: 2 of 27 | n.r. | T1: 5-yr 92  T1: 10-yr 74 T2: 5-yr 87 T2: 10-yr 75 |
| *Wykypiel* | 15 | T1 (n=15) | T1: 3 of 15 | | T1: 3 of 15 | | T1: 0 of 15 | n.r. | n.r. |
| *Budhoo* | 22 | T1 (n=10) T2 (n=12) | T1: 0 of 10 T2: 6 of 12 | | T1: 0 of 10 T2: 5 of 12 | | T1: 0 of 10 T2: 1 of 12 | n.r. | T1: 5-yr 73 |
| *Garcia-Aguilar* | 82 | T1 (n=55) T2 (n=27) | T1: 11 of 55 T2: 10 of 27 | | T1: 10 of 55 T2: 10 of 27 | | T1: 2 of 55 T2: 2 of 27 | T1 77 T2 55 | T1: 98 T2: 89 |
| *Lamont* | 23 | T1 (n=17) T2 (n=6) | T1: 4 of 17 T2: 0 of 6 | | T1: 4 of 17 T2: 0 of 6 | | T1: 0 of 17 T2: 0 of 6 | n.r. | n.r. |
| *Mellgren* | 108 | T1 (n=69) T2 (n=39) | T1: 12 of 69 T2: 18 of 39 | | T1: 12 of 69 T2: 18 of 39 | | n.r. | n.r. | T1: 5-yr: 72 T2: 5-yr: 65 |
| *Russell* | 14 | T1 (n=14) | T1: 2 of 14 | | T1: 1 of 14 | | T1: 1 of 14 | n.r. | n.r. |
| *Chakravarti* | 52 | T1 (n=44)  T2 (n=8) | T1: 5 of 44  T2: 5 of 8 | | T1: 5 of 44  T2: 5 of 8 | | n.r. | n.r. | n.r. |
| *Heintz* | 58 | T1 (n=58) | T1: 6 of 58 | | T1: 6 of 58 | | n.r. | n.r. | n.r. |
| *Taylor* | 23 | T1 (n=15) T2 (n= 8) | T1: 6 of 15 T2: 4 of 8 | | T1: 6 of 15 T2: 4 of 8 | | T1: 2 of 15 T2: 0 of 8 | n.r. | n.r. |
| *Bleday* | 21 | T1 (n=21) | T1: 2 of 21 | | T1: 2 of 21 | | n.r. | n.r. | n.r. |
| *Winde* | 25 | T1 (n=25) | T1: 1 of 25 | | T1: 1 of 25 | | n.r. | n.r. | T1: 5-yr: 96 |
| *Coco* | 22 | T1 (n=22) | T1: 1 of 22 | | T1: 1 of 22 | | T1: 0 of 22 | n.r. | n.r. |

DFS: disease-free survival, OS: overall survival, n.r,: not reported

**Table S6 Outcome data of local excision followed by completion total mesorectal excision**

| *Reference* | *Size of cohort* | *pT category* | Overall recurrence | Overall local recurrence | Overall distant recurrence | DFS % | OS % |
| --- | --- | --- | --- | --- | --- | --- | --- |
| *Antonelli* | 13 | T1 (n=13) | T1: 0 of 13 | T1: 0 of 13 | T1: 0 of 13 | n.r. | n.r. |
| *Ortenzi* | 30 | T1 (n=15) T2 (n=15) | T1: 0 of 15 T2: 0 of 15 | T1: 0 of 15 T2: 0 of 15 | n.r. | n.r. | n.r. |
| *Tamaru* | 56 | T1 (n=56) | T1: 3 of 56 | T1: 2 of 56 | T1: 3 of 56 | n.r. | n.r. |
| *Morino* | 5 | T2 (n=5) | T2: 1 of 5 | T2: 1 of 5 | T2: 0 of 5 | n.r. | n.r. |
| *Choi* | 13 | T1 (n=13) | T1: 0 of 13 | T1: 0 of 13 | T1: 0 of 13 | T1: 100 | n.r. |
| *Borschitz* | 39 | T1 (n=19)  T2 (n=20) | T1: 3 of 19 T2: 4 of 20 | T1: 1 of 19 T2: 2 of 20 | T1: 2 of 19 T2: 2 of 20 | T1-2: 10-yr 86 | n.r. |
| *Duek* | 5 | T2 (n=5) | T2: 0 of 5 | T2: 0 of 5 | T2: 0 of 5 | T2: 3-yr 100 | n.r. |
| *Min* | 7 | T2 (n=7) | T2: 1 of 7 | T2: 0 of 7 | T2: 1 of 7 | n.r. | n.r. |
| *Lee, WY* | 9 | T1 (n=3)  T2 (n=6) | T1: 0 of 3 T2: 0 of 6 | T1: 0 of 3 T2: 0 of 6 | T1: 0 of 3 T2: 0 of 6 | n.r. | n.r. |
| *Hahnloser* | 37 | T1 (n=29) T2 (n=8) | T1: 2 of 29 T2: 1 of 8 | T1: 0 of 29 T2: 0 of 8 | T1: 2 of 29 T2: 1 of 8 | T1-2: 5-yr 94 T1-2: 10-yr 90 | T1-2: 5-yr 79 T1-2: 10-yr 62 |
| *Nakagoe* | 11 | T1 (n= 8)  T2 (n=3) | T1: 0 of 8 T2: 0 of 3 | T1: 0 of 8 T2: 0 of 3 | T1: 0 of 8 T2: 0 of 3 | n.r. | n.r. |
| *Wykypiel* | 3 | T1 (n=2)  T2 (n=1) | T1: 0 of 2  T2: 0 of 1 | T1: 0 of 2  T2: 0 of 1 | T1: 0 of 2  T2: 0 of 1 | n.r. | n.r. |
| *Heintz* | 22 | T1 (n=22) | T1: 3 of 22 | T1: 2 of 22 | T1: 1 of 22 | n.r. | n.r. |

DFS: disease-free survival, OS: overall survival, n.r,: not reported

**Table S7 Outcome data of local excision followed by adjuvant (chemo)radiation**

| *Reference* | *Size of cohort* | *pT category* | Overall recurrence | Overall local recurrence | Overall distant recurrence | DFS % | OS % |
| --- | --- | --- | --- | --- | --- | --- | --- |
| *Jones* | 26 | T1 (n=8)  T2 (n=18) | T1: 2 of 8  T2: 2 of 18 | T1: 2 of 8  T2: 1 of 18 | T1: 1 of 8  T2: 1 of 18 | n.r. | n.r. |
| *Suzuki* | 50 | T1 (n=50) | T1: 4 of 50 | T1: 1 of 50 | T1: 3 of 50 | T1: 5-yr 86 | T1: 5- yr 96 |
| *Balyasnikova* | 18 | T1 (n=11) T2 (n=7) | T1: 0 of 11 T2: 0 of 7 | T1: 0 of 11 T2: 0 of 7 | T1: 0 of 11 T2: 0 of 7 | T1: 3-yr 100 T2: 3-yr 100 | T1: 4-yr 100 T2: 4-yr 100 |
| *O’Neill* | 21 | T1 (n=4) T2 (n=17) | T1: 0 of 4 T2: 2 of 17 | T1: 0 of 4 T2: 0 of 17 | T1: 0 of 4 T2: 2 of 17 | T1: 3-yr 100 T2: 3-yr 87.1 | T1: 3-yr 100 T2: 3-yr 100 |
| *Sasaki* | 57 | T1 (n=53) T2 (n=4) | T1: 1 of 53 T2: 1 of 4 | T1: 1 of 53 T2: 1 of 4 | T1: 1 of 53 T2: 0 of 4 | T1: 5-yr 94 T2: 5-yr 75 | T1: 5-yr 98 T2: 5-yr n.r. |
| *Jeong* | 83 | T1 (n=68) T2 (n=15) | T1: 2 of 68 T2: 3 of 15 | T1: 1 of 68 T2: 2 of 15 | T1: 2 of 68 T2: 1 of 15 | T1: 5-yr 93.8 T2: 5-yr 73.3 | T1: 5-yr 95.2 T2: 5-yr 93.3 |
| *Rackley* | 88 | T1 (n=46) T2 (n=42) | T1: 3 of 46 T2: 9 of 42 | T1: 3 of 46 T2: 9 of 42 | n.r. | T1: 5-yr 90.0 T2: 5-yr 78.2 | T1: 5-yr 84.3 T2: 5-yr 79.2 |
| *Lee, S* | 31 | T1 (n=31 | T1: 2 of 31 | T1: 2 of 31 | T1: 2 of 31 | T1: 5-yr 96.8 | n.r. |
| *Bacic* | 4 | T2 (n=4) | T2: 2 of 4 | T2: 2 of 4 | T2: 0 of 4 | n.r. | n.r. |
| *Sun* | 49 | T1 (n=8) T2 (n=41) | T1: 0 of 8 T2: 3 of 41 | T1: 0 of 8 T2: 3 of 41 | n.r. | T1: 100 T2: n.r. | T1: 5-yr 63 T1: 10-yr 50 T2: 5-yr 61 T2: 10-yr 34 |
| *Ramirez* | 28 | T1 (n=6) T2 (n=22) | T1: 1 of 6 T2: 2 of 22 | T1 1 of 6 T2: 2 of 22 | T1: 0 of 6 T2: 0 of 22 | n.r. | n.r. |
| *Morino* | 19 | T2 (n=19) | T2: 4 of 19 | T2: 4 of 19 | T2: 0 of 19 | n.r. | n.r. |
| *Tsai* | 5 | T2 (n=5) | T2: 1 of 5 | T2: 1 of 5 | T2: 0 of 5 | n.r. | n.r. |
| *Allaix* | 19 | T1 (n=3)  T2 (n=16) | T1: 0 of 3  T2: 4 of 16 | n.r. | n.r. | n.r. | n.r. |
| *Greenberg* | 51 | T2 (n=51) | T2: 12 of 51 | T2: 9 of 51 | T2: 6 of 51 | T2: 10-yr 64 | T2: 10-yr 66 |
| *Duek* | 12 | T2 (n=12) | T2: 0 of 12 | T2: 0 of 12 | T2: 0 of 12 | T2: 3-yr 100 | n.r. |
| *Min* | 19 | T1 (n=11) T2 (n=8) | T1: 2 of 11 T2: 1 of 8 | T1: 0 of 11 T2: 1 of 8 | T1: 2 of 11 T2: 0 of 8 | n.r. | n.r. |
| *Ganai* | 1 | T1 (n=1) | T1: 0 of 1 | T1: 0 of 1 | T1: 0 of 1 | n.r. | n.r. |
| *Stipa* | 6 | T1 (n=2) T2 (n=4) | T1: 0 of 2 T2: 2 of 4 | T1: 0 of 2 T2: 0 of 4 | T1: 0 of 2 T2: 2 of 4 | n.r. | n.r. |
| *Gopaul* | 15 | T1 (n= 4) T2 (n= 11) | T1: 1 of 4 T2: 1 of 11 | T1: 1 of 4 T2: 1 of 11 | n.r. | n.r. | n.r. |
| *Paty* | 31 | T1 (n=7)  T2 (n=24) | T1: 3 of 7  T2: 8 of 24 | T1: 1 of 7  T2: 6 of 24 | T1: 2 of 7  T2: 2 of 24 | n.r. | T1: 10-yr 71  T2: 10-yr 68 |
| *Benson* | 60 | T1 (n=24)  T2 (n=36) | T1: 9 of 24  T2: 8 of 36 | T1: 9 of 24  T2: 8 of 36 | n.r. | T1: 5-yr 59  T2: 5-yr 58 | T1: 5-yr 76  T2: 5-yr 58 |
| *Lamont* | 20 | T1 (n=10) T2 (n=10) | T1: 0 of 10 T2: 2 of 10 | T1: 0 of 10 T2: 2 of 10 | T1: 0 of 10 T2: 2 of 10 | T1: 100 T2: 70 | n.r. |
| *Chakravarti* | 47 | T1 (n=13)  T2 (n=33) | T1: 0 of 14  T2: 5 of 33 | T1: 0 of 14  T2: 5 of 33 | n.r. | n.r. | n.r. |
| *Wagman* | 31 | T1 (n=6) T2 (n=25) | T1: 0 of 6 T2: 6 of 25 | T1: 0 of 6 T2: 6 of 25 | T1: 0 of 6 T2: 0 of 25 | n.r. | n.r. |
| *Taylor* | 21 | T1 (n=12) T2 (n=9) | T1: 1 of 12 T2: 1 of 9 | T1: 1 of 12 T2: 0 of 9 | T1: 1 of 12 T2: 1 of 9 | T1-2: 5-yr 81 | n.r. |
| *Valentini* | 21 | T1 (n=9)  T2 (n=12) | T1: 1 of 9  T2: 2 of 12 | T1: 1 of 9  T2: 2 of 12 | n.r. | n.r. | n.r. |
| *Coco* | 15 | T2 (n=15) | T2: 1 of 15 | T2: 1 of 15 | n.r. | n.r. | T2: 5-yr 90 |

DFS: disease-free survival, OS: overall survival, n.r,: not reported

**Table S8 Outcome data of local excision without additional treatment, subgroup analysis low- and high-risk pT1.**

| *Reference* | *Size of cohort* | *LR vs HR* | Overall recurrence | Overall local recurrence | | Overall distant recurrence | | DFS % | OS % |
| --- | --- | --- | --- | --- | --- | --- | --- | --- | --- |
| *O’neill* | 50 | LR (n=50) | LR 4 of 50 | | LR 2 of 50 | | LR 2 of 50 | LR 3-yr 94.3 | LR 3-yr 81.8 |
| *Balyasnikova* | 6 | LR (n=6) | LR 0 of 6 | | LR 0 of 6 | | LR 0 of 6 | n.r. | LR 4-yr 100 |
| *Tamaru* | 29 | HR (n=29) | HR 1 of 29 | | HR 1 of 29 | | HR 0 of 29 | n.r. | n.r. |
| *Restivo* | 32 | LR (n=32) | LR 1 of 32 | | LR 1 of 32 | | n.r. | n.r. | n.r. |
| *Junginger* | 86 | LR (n=64)  HR (n=22) | LR 7 of 64  HR 7 of 22 | | LR 6 of 64  HR 7 of 22 | | LR 1 of 64  HR n.r. | n.r. | n.r. |
| *Turza* | 21 | LR (n=14)  HR (n=7) | LR 1 of 14  HR 0 of 7 | | LR 1 of 14  HR 0 of 7 | | LR 0 of 14  HR 0 of 7 | n.r. | n.r. |
| *Lee, S* | 35 | HR (n=35) | HR 4 of 35 | | HR 2 of 35 | | HR 2 of 35 | HR 5-yr 97 | n.r. |
| *Ikematsu* | 53 | LR (n=16)  HR (n=37) | LR 1 of 16  HR 6 of 37 | | LR 0 of 16  HR 4 of 37 | | LR 1 of 16  HR 2 of 37 | LR 5-yr 90.0  HR 5-yr 77.7 | LR 5-yr 88.9  HR 5-yr 96.2 |
| *Amann* | 39 | LR (n=39) | LR 4 of 39 | | LR 4 of 39 | | LR 1 of 39 | LR 97.6 | n.r. |
| *Morino* | 45 | LR (n=38)  HR (n=10) | LR 2 of 38  HR 3 of 10 | | LR 2 of 38  HR 3 of 10 | | LR 0 of 38  HR 1 of 10 | HR 5-yr 63 | n.r. |
| *Ramirez* | 53 | LR (n=53) | LR 4 of 53 | | LR 3 of 53 | | LR 1 of 53 | n.r. | n.r. |
| *Peng* | 58 | LR (n=43)  HR (n=15) | LR 0 of 43  HR 6 of 15 | | LR 0 of 43  HR 6 of 15 | | n.r. | n.r. | n.r. |
| *Huh* | 22 | LR (n=22) | LR 6 of 22 | | LR 5 of 22 | | LR 1 of 22 | LR 5-yr 95.9 | LR 5-yr 100 |
| *Lebedyev* | 37 | LR (n=4)  HR (n=33) | LR 0 of 6  HR 2 of 33 | | LR 0 of 6  HR 2 of 33 | | LR 0 of 6  HR 1 of 33 | LR 100  HR 91 | n.r. |
| *Borschitz* | 117 | LR (n=93)  HR (n=24) | LR 8 of 93  HR 13 of 24 | | LR 6 of 93  HR 9 of 24 | | LR 2 of 93  HR 4 of 24 | n.r. | n.r. |
| *Serra-Aracil* | 17 | LR (n=16)  HR (n=1) | LR 1 0f 16  HR 0 of 1 | | LR 1 of 16  HR 0 of 1 | | LR 0 of 16  HR 0 of 1 | LR 92.3  HR 100 | LR 100  HR 100 |
| *Lee, WY* | 6 | HR (n=6) | HR 1 of 6 | | HR 1 of 6 | | HR 1 of 6 | n.r. | n.r. |
| *Min* | 26 | LR (n=26) | LR 4 of 26 | | LR 4 of 26 | | LR 1 of 26 | n.r. | n.r. |
| *Ptok* | 99 | LR (n=99) | LR 9 of 99 | | LR 5 of 99 | | LR 4 of 99 | LR 5-yr 91.4 | LR 5-yr 83.6 |
| *Ganai* | 19 | LR (n=18)  HR (n=1) | LR 2 of 18  HR 0 of 1 | | LR 2 of 18  HR 0 of 1 | | LR 0 of 18  HR 0 of 1 | n.r. | n.r. |
| *Maskelar* | 27 | LR (n=23)  HR (n=4) | LR 0 of 23  HR 0 of 4 | | LR 0 of 23  HR 0 of 4 | | LR 0 of 23  HR 0 of 4 | n.r. | n.r. |
| *Nascimbeni* | 70 | LR (n=40)  HR (n=30) | LR 6 of 40  HR 11 of 30 | | LR 2 of 40  HR 4 of 30 | | LR 4 of 40  HR 7 of 30 | n.r. | n.r. |
| *Araki* | 22 | LR (n=18)  HR (n= 4) | LR 0 of 18  HR 0 of 4 | | LR 0 of 18  HR 0 of 4 | | LR 0 of 18  HR 0 of 4 | n.r. | n.r. |
| *Lee, W* | 52 | LR (n=52) | LR 2 of 52 | | LR 2 of 52 | | n.r. | LR 5-yr 95.9 | LR 5-yr 100 |
| *Nakagoe* | 15 | LR (n=12)  HR (n=3) | LR 0 of 12  HR 0 of 3 | | LR 0 of 12  HR 0 of 3 | | LR 0 of 12  HR 0 of 3 | n.r. | n.r. |
| *Paty* | 70 | LR (n=63)  HR (n=7) | LR 8 of 63  HR 3 of 7 | | LR 8 of 63  HR 1 of 7 | | LR n.r.  HR 2 of 7 | n.r. | LR 10-yr 87  HR 10-yr 71 |
| *Wykypiel* | 15 | LR (n=15) | LR 3 of 15 | | LR 3 of 15 | | LR 0 of 15 | n.r. | n.r. |
| *Garcia-Aguilar* | 55 | LR (n=55) | LR 11 of 55 | | LR 10 of 55 | | LR 2 of 55 | LR 4.5-yr 77 | LR 4.5-yr 98 |
| *Lamont* | 17 | LR (n=15)  HR (n= 2) | LR 4 of 15  HR 0 of 2 | | LR 4 of 15  HR 0 of 2 | | LR 2 of 15  HR 0 of 2 | n.r. | n.r. |
| *Russel* | 14 | LR (n=14) | LR 2 of 14 | | LR 1 of 14 | | LR 1 of 14 | n.r. | n.r. |
| *Heintz* | 58 | LR (n=46)  HR (n=12) | LR 2 of 46  HR 4 of 12 | | LR 2 of 46  HR 4 of 12 | | n.r. | n.r. | n.r. |
| *Winde* | 25 | LR (n=25) | LR 1 of 25 | | LR 1 of 25 | | LR 0 of 25 | n.r. | LR 5-yr 96 |
| *Coco* | 22 | LR (n=22) | LR 1 of 22 | | LR 1 of 22 | | LR 0 of 22 | n.r. | n.r. |

DFS: disease-free survival, OS: overall survival, n.r,: not reported, LR: low-risk, HR: high-risk

**Table S9 Outcome data of local excision followed by completion total mesorectal excision, subgroup analysis low- and high-risk pT1**

| *Reference* | *Size of cohort* | *LR vs HR* | Overall recurrence | Overall local recurrence | | Overall distant recurrence | | DFS % | OS % |
| --- | --- | --- | --- | --- | --- | --- | --- | --- | --- |
| *Ortenzi* | 15 | HR (n=15) | HR 0 of 15 | | HR 0 of 15 | | n.r. | n.r. | n.r. |
| *Tamaru* | 56 | HR (n=56) | HR 3 of 56 | | HR 2 of 56 | | HR 3 of 56 | n.r. | n.r. |
| *Borschitz* | 19 | HR (n=19) | HR 3 of 19 | | HR 1 of 19 | | HR 2 of 19 | n.r. | n.r. |
| *Lee, WY* | 3 | HR (n=3) | HR 0 of 3 | | HR 0 of 3 | | HR 0 of 3 | n.r. | n.r. |
| *Nakagoe* | 8 | HR (n=8) | HR 0 of 8 | | HR 0 of 8 | | HR 0 of 8 | n.r. | n.r. |
| *Heintz* | 7 | HR (n=7) | HR 2 of 7 | | HR 2 of 7 | | HR 1 0f 7 | n.r. | HR 5-yr 69 |
| *Winde* | 28 | LR (n=28) | LR 1 of 28 | | LR 0 of 28 | | LR 1 of 28 | n.r. | LR 5-yr 96 |

DFS: disease-free survival, OS: overall survival, n.r,: not reported, LR: low-risk, HR: high-risk

**Table S10 Outcome data of local excision followed by adjuvant (chemo)radiotherapy, subgroup analysis low- and high-risk pT1**

| *Reference* | *Size of cohort* | *LR vs HR* | Overall recurrence | Overall local recurrence | | Overall distant recurrence | | DFS % | OS % |
| --- | --- | --- | --- | --- | --- | --- | --- | --- | --- |
| *Jones* | 8 | LR (n=1)  HR (n=7) | LR 0 of 1  HR 2 of 7 | | LR 0 of 1  HR 2 of 7 | | LR 0 of 1  HR 1 of 7 | n.r. | n.r. |
| *Balyasnikova* | 11 | HR (n=11) | HR 0 of 11 | | HR 0 of 11 | | HR 0 of 11 | HR 3-yr 100 | HR 4-yr 100 |
| *O’Neill* | 4 | HR (n=4) | HR 0 of 4 | | HR 0 of 4 | | HR 0 of 4 | HR 3-yr 100 | HR 3-yr 100 |
| *Sasaki* | 53 | HR (n=53) | HR 1 of 53 | | HR 1 of 53 | | HR 1 of 53 | HR 5-yr 94 | HR 5-yr 98 |
| *Jeong* | 68 | HR (n=68) | HR 2 of 68 | | HR 1 of 68 | | HR 2 of 68 | HR 5-yr 93.8 | HR 5-yr 95.2 |
| *Rackley* | 46 | HR (n=46) | HR 3 of 46 | | HR 3 of 46 | | n.r. | HR 5-yr 90.0 | HR 5-yr 84.3 |
| *Lee, S* | 31 | HR (n=31) | HR 2 of 31 | | HR 2 of 31 | | HR 2 of 31 | HR T1 5-yr 96.8 | n.r. |
| *Ramirez* | 6 | HR (n=6) | HR 1 of 6 | | HR 1 of 6 | | HR 0 of 6 | n.r. | n.r. |
| *Min* | 11 | HR (n=11) | HR 2 of 11 | | HR 0 of 11 | | HR 2 of 11 | n.r. | n.r. |
| *Ganai* | 1 | HR (n=1) | HR 0 of 1 | | HR 0 of 1 | | HR 0 of 1 | n.r. | n.r. |
| *Lamont* | 10 | HR (n=10) | HR 0 of 10 | | HR 0 of 10 | | HR 0 of 10 | n.r. | HR T1 100 |
| *Wagman* | 6 | HR (n=6) | HR 0 of 6 | | HR 0 of 6 | | HR 0 of 6 | n.r. | n.r. |

DFS: disease-free survival, OS: overall survival, n.r,: not reported, LR: low-risk, HR: high-risk

**Table S11 Quality assessment of studies on no additional treatment after local excision**

| **Author** | **Aim** | **Inclusion** | **Prospective** | **Endpoints** | **Unbiased assessment** | **Follow-up period** | **Loss to follow up** | **Sample size** | **Allocation bias** | **Total** |
| --- | --- | --- | --- | --- | --- | --- | --- | --- | --- | --- |
| **Kwakye** | 2 | 2 | 0 | 2 | 0 | 2 | 1 | 0 | 0 | 9 |
| **Kouyama** | 2 | 2 | 0 | 2 | 0 | 2 | 1 | 0 | 1 | 10 |
| **Jones** | 2 | 2 | 1 | 2 | 2 | 2 | 0 | 0 | 1 | 12 |
| **Balyasnikova** | 2 | 2 | 0 | 2 | 2 | 2 | 1 | 0 | 2 | 13 |
| **O'Neill** | 2 | 2 | 0 | 2 | 0 | 2 | 0 | 0 | 2 | 10 |
| **Tamaru** | 2 | 2 | 0 | 2 | 2 | 2 | 2 | 0 | 2 | 14 |
| **Junginger** | 2 | 2 | 1 | 2 | 1 | 2 | 2 | 0 | 2 | 14 |
| **Restivo** | 2 | 2 | 1 | 2 | 2 | 2 | 2 | 0 | 2 | 15 |
| **Stornes** | 2 | 2 | 1 | 2 | 2 | 1 | 0 | 0 | 2 | 12 |
| **Turza** | 2 | 0 | 1 | 2 | 2 | 2 | 0 | 0 | 0 | 9 |
| **Amann** | 2 | 1 | 0 | 2 | 1 | 2 | 1 | 0 | 0 | 9 |
| **Lee, S** | 2 | 2 | 0 | 2 | 2 | 2 | 0 | 0 | 2 | 12 |
| **Bacic** | 1 | 2 | 0 | 2 | 0 | 2 | 0 | 0 | 1 | 8 |
| **Elmessiry** | 2 | 1 | 1 | 2 | 0 | 2 | 0 | 0 | 1 | 9 |
| **Guerrieri** | 2 | 1 | 0 | 2 | 2 | 2 | 2 | 0 | 1 | 12 |
| **Sun** | 1 | 0 | 0 | 1 | 2 | 2 | 2 | 0 | 0 | 8 |
| **Ikematsu** | 2 | 2 | 0 | 2 | 2 | 2 | 0 | 0 | 2 | 12 |
| **Im** | 2 | 2 | 0 | 2 | 1 | 2 | 0 | 0 | 2 | 11 |
| **Luglio** | 2 | 1 | 1 | 2 | 0 | 2 | 0 | 0 | 2 | 10 |
| **Amann** | 1 | 2 | 0 | 2 | 2 | 1 | 2 | 0 | 0 | 10 |
| **Morino** | 2 | 2 | 1 | 2 | 0 | 2 | 1 | 0 | 1 | 11 |
| **Oka** | 2 | 0 | 0 | 2 | 0 | 2 | 0 | 0 | 1 | 7 |
| **Ramirez** | 2 | 2 | 2 | 2 | 2 | 2 | 2 | 0 | 2 | 16 |
| **Doornebosch** | 2 | 2 | 0 | 2 | 2 | 2 | 0 | 0 | 2 | 12 |
| **Peng** | 2 | 1 | 0 | 2 | 2 | 2 | 0 | 0 | 0 | 9 |
| **Tsai** | 2 | 2 | 1 | 2 | 2 | 2 | 1 | 0 | 2 | 14 |
| **Allaix** | 2 | 1 | 1 | 2 | 1 | 2 | 2 | 0 | 1 | 12 |
| **Choi** | 2 | 1 | 1 | 2 | 1 | 2 | 2 | 0 | 2 | 13 |
| **Huh** | 2 | 2 | 0 | 2 | 2 | 2 | 0 | 0 | 1 | 11 |
| **Lebedyev** | 2 | 1 | 0 | 2 | 2 | 2 | 2 | 0 | 2 | 13 |
| **Borschitz** | 2 | 2 | 0 | 2 | 1 | 2 | 0 | 0 | 2 | 11 |
| **Duek** | 2 | 2 | 0 | 2 | 2 | 2 | 0 | 0 | 2 | 12 |
| **Greenberg** | 2 | 1 | 0 | 2 | 2 | 2 | 2 | 1 | 2 | 14 |
| **Serra-Aracil** | 2 | 1 | 2 | 2 | 2 | 2 | 2 | 0 | 2 | 15 |
| **Lee, WY** | 2 | 1 | 0 | 2 | 1 | 2 | 0 | 0 | 2 | 10 |
| **Min** | 2 | 0 | 0 | 2 | 2 | 2 | 2 | 0 | 2 | 12 |
| **Ptok** | 2 | 2 | 2 | 2 | 0 | 2 | 2 | 0 | 0 | 12 |
| **You** | 2 | 0 | 0 | 2 | 0 | 2 | 2 | 0 | 0 | 8 |
| **Zacharakis** | 2 | 2 | 1 | 2 | 2 | 2 | 0 | 0 | 1 | 12 |
| **Floyd** | 2 | 1 | 0 | 2 | 1 | 1 | 0 | 0 | 2 | 9 |
| **Ganai** | 2 | 2 | 1 | 2 | 1 | 2 | 0 | 0 | 1 | 11 |
| **Maslekar** | 2 | 1 | 2 | 1 | 2 | 2 | 0 | 0 | 1 | 11 |
| **Endreseth** | 1 | 2 | 0 | 2 | 0 | 1 | 2 | 0 | 0 | 8 |
| **Gopaul** | 2 | 2 | 0 | 2 | 2 | 2 | 0 | 0 | 0 | 10 |
| **Nascimbeni** | 2 | 2 | 0 | 2 | 0 | 2 | 0 | 0 | 0 | 8 |
| **Araki** | 2 | 1 | 0 | 1 | 1 | 2 | 2 | 0 | 2 | 11 |
| **Gonzalez** | 1 | 2 | 0 | 1 | 0 | 2 | 0 | 0 | 2 | 8 |
| **Lee, W** | 2 | 2 | 0 | 2 | 0 | 2 | 2 | 0 | 1 | 11 |
| **Nakagoe** | 2 | 2 | 1 | 1 | 1 | 2 | 2 | 0 | 2 | 13 |
| **Paty** | 2 | 2 | 1 | 2 | 0 | 2 | 0 | 0 | 2 | 11 |
| **Wykypiel** | 2 | 2 | 0 | 2 | 0 | 2 | 0 | 0 | 2 | 10 |
| **Budhoo** | 1 | 2 | 0 | 1 | 1 | 2 | 1 | 0 | 1 | 9 |
| **Garcia-Aguilar** | 2 | 2 | 0 | 2 | 1 | 2 | 0 | 0 | 1 | 10 |
| **Lamont** | 2 | 1 | 0 | 2 | 2 | 2 | 0 | 0 | 0 | 9 |
| **Mellgren** | 2 | 2 | 0 | 2 | 1 | 2 | 1 | 0 | 0 | 10 |
| **Russell** | 2 | 2 | 2 | 1 | 2 | 2 | 0 | 0 | 2 | 13 |
| **Chakravarti** | 2 | 0 | 0 | 2 | 1 | 2 | 0 | 0 | 0 | 7 |
| **Heintz** | 1 | 2 | 0 | 2 | 2 | 2 | 2 | 0 | 2 | 13 |
| **Taylor** | 1 | 0 | 0 | 1 | 1 | 2 | 2 | 0 | 0 | 7 |
| **Bleday** | 1 | 1 | 2 | 2 | 1 | 2 | 0 | 0 | 2 | 11 |
| **Winde** | 2 | 2 | 2 | 2 | 2 | 2 | 0 | 0 | 2 | 14 |
| **Coco** | 1 | 1 | 0 | 1 | 1 | 1 | 0 | 0 | 2 | 7 |
| **Not reported** | 0 | 7 | 40 | 0 | 18 | 0 | 33 | 61 | 15 | 174 |
| **Reported but inadequate** | 10 | 17 | 15 | 9 | 16 | 5 | 8 | 1 | 15 | 96 |
| **Reported but adequate** | 52 | 38 | 7 | 53 | 28 | 57 | 21 | 0 | 32 | 288 |

Quality assessment close surveillance following local excision according to the Methodological Index for Non-Randomized Studies (MINORS) checklist. 0, Not reported; 1, reported but inadequate; 2, reported but adequate

**Table S12 Quality assessment of studies on completion total mesorectal excision and adjuvant (chemo)radiation following local excision**

| **Author** | **Aim** | **Inclusion** | **Prospective** | **Endpoints** | **Unbiased assessment** | **Follow-up period** | **Loss to follow up** | **Sample size** | **Allocation bias** | **Total** |
| --- | --- | --- | --- | --- | --- | --- | --- | --- | --- | --- |
| **Antonelli** | 2 | 2 | 0 | 2 | 2 | 2 | 0 | 0 | 1 | 11 |
| **Jones** | 1 | 2 | 1 | 2 | 2 | 2 | 1 | 0 | 2 | 13 |
| **Ortenzi** | 1 | 1 | 0 | 1 | 1 | 2 | 1 | 0 | 2 | 9 |
| **Suzuki** | 1 | 2 | 0 | 2 | 2 | 2 | 1 | 0 | 2 | 12 |
| **Balyasnikova** | 2 | 2 | 0 | 2 | 2 | 2 | 1 | 0 | 2 | 13 |
| **O'Neill** | 2 | 2 | 0 | 2 | 0 | 2 | 0 | 0 | 2 | 10 |
| **Sasaki** | 2 | 2 | 2 | 2 | 0 | 2 | 0 | 0 | 2 | 12 |
| **Tamaru** | 2 | 2 | 0 | 2 | 2 | 2 | 2 | 0 | 2 | 14 |
| **Jeong** | 2 | 2 | 0 | 2 | 2 | 2 | 0 | 0 | 0 | 10 |
| **Rackley** | 2 | 2 | 0 | 2 | 2 | 1 | 0 | 0 | 1 | 10 |
| **Lee, S** | 2 | 2 | 0 | 2 | 2 | 2 | 0 | 0 | 2 | 12 |
| **Bacic** | 1 | 2 | 0 | 2 | 0 | 2 | 0 | 0 | 1 | 8 |
| **Sun** | 1 | 0 | 0 | 1 | 2 | 2 | 2 | 0 | 0 | 8 |
| **Ramirez** | 2 | 2 | 2 | 2 | 2 | 2 | 2 | 0 | 2 | 16 |
| **Morino** | 2 | 1 | 1 | 2 | 2 | 2 | 0 | 0 | 0 | 10 |
| **Tsai** | 2 | 2 | 1 | 2 | 2 | 2 | 1 | 0 | 2 | 14 |
| **Allaix** | 2 | 1 | 1 | 2 | 1 | 2 | 2 | 0 | 1 | 12 |
| **Choi** | 2 | 1 | 1 | 2 | 1 | 2 | 2 | 0 | 2 | 13 |
| **Greenberg** | 2 | 1 | 0 | 2 | 2 | 2 | 2 | 1 | 2 | 14 |
| **Borschitz** | 2 | 2 | 0 | 2 | 2 | 2 | 0 | 0 | 2 | 12 |
| **Duek** | 2 | 2 | 0 | 2 | 2 | 2 | 0 | 0 | 2 | 12 |
| **Min** | 2 | 0 | 0 | 2 | 2 | 2 | 2 | 0 | 2 | 12 |
| **Lee, WY** | 2 | 1 | 0 | 2 | 1 | 2 | 0 | 0 | 2 | 10 |
| **Ganai** | 2 | 2 | 1 | 2 | 1 | 2 | 0 | 0 | 1 | 11 |
| **Stipa** | 1 | 2 | 0 | 2 | 2 | 2 | 0 | 0 | 0 | 9 |
| **Hahnloser** | 2 | 2 | 0 | 2 | 2 | 1 | 2 | 0 | 2 | 13 |
| **Gopaul** | 2 | 2 | 0 | 2 | 2 | 2 | 0 | 0 | 0 | 10 |
| **Nakagoe** | 2 | 2 | 0 | 2 | 2 | 2 | 2 | 0 | 2 | 14 |
| **Paty** | 2 | 2 | 1 | 2 | 0 | 2 | 0 | 0 | 2 | 11 |
| **Wykypiel** | 2 | 2 | 0 | 2 | 0 | 2 | 0 | 0 | 2 | 10 |
| **Benson** | 1 | 1 | 0 | 2 | 1 | 2 | 1 | 0 | 1 | 9 |
| **Lamont** | 2 | 1 | 0 | 2 | 2 | 2 | 0 | 0 | 0 | 9 |
| **Chakravarti** | 2 | 0 | 0 | 2 | 1 | 2 | 0 | 0 | 0 | 7 |
| **Wagman** | 2 | 0 | 2 | 2 | 2 | 2 | 1 | 0 | 2 | 13 |
| **Taylor** | 1 | 0 | 0 | 1 | 1 | 2 | 2 | 0 | 0 | 7 |
| **Heintz** | 1 | 2 | 0 | 2 | 2 | 2 | 2 | 0 | 2 | 13 |
| **Valentini** | 1 | 2 | 0 | 2 | 1 | 2 | 2 | 0 | 0 | 10 |
| **Coco** | 1 | 1 | 0 | 1 | 1 | 1 | 0 | 0 | 2 | 7 |
| **Not reported** | 0 | 5 | 31 | 0 | 5 | 0 | 20 | 25 | 10 | 96 |
| **Reported but inadequate** | 13 | 10 | 8 | 5 | 13 | 4 | 9 | 1 | 6 | 69 |
| **Reported but adequate** | 29 | 27 | 3 | 37 | 24 | 38 | 13 | 0 | 26 | 197 |

Quality assessment of adjuvant (chemo)radiation and completion total mesorectal excision following local excision according to the Methodological Index for Non-Randomized Studies (MINORS) checklist. 0, Not reported; 1, reported but inadequate; 2, reported but adequate

**Figure S1** **Forest plots of overall local recurrence of local without additional treatment in patients with a) pT1 and b) pT2 tumours. An inverse-variance random-effects model. Proportions with 95 per cent confidence intervals**

**a pT1**


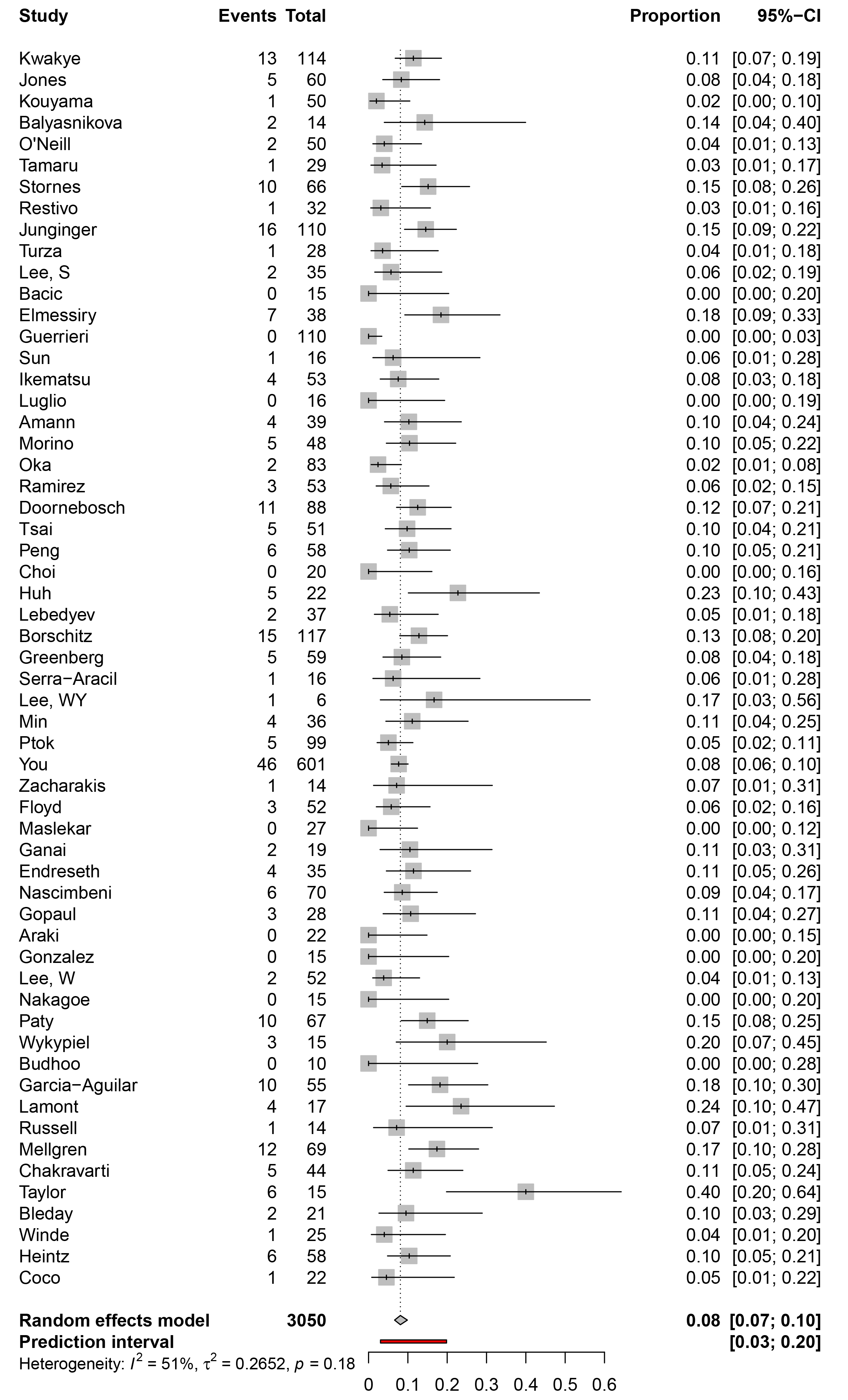


b pT2


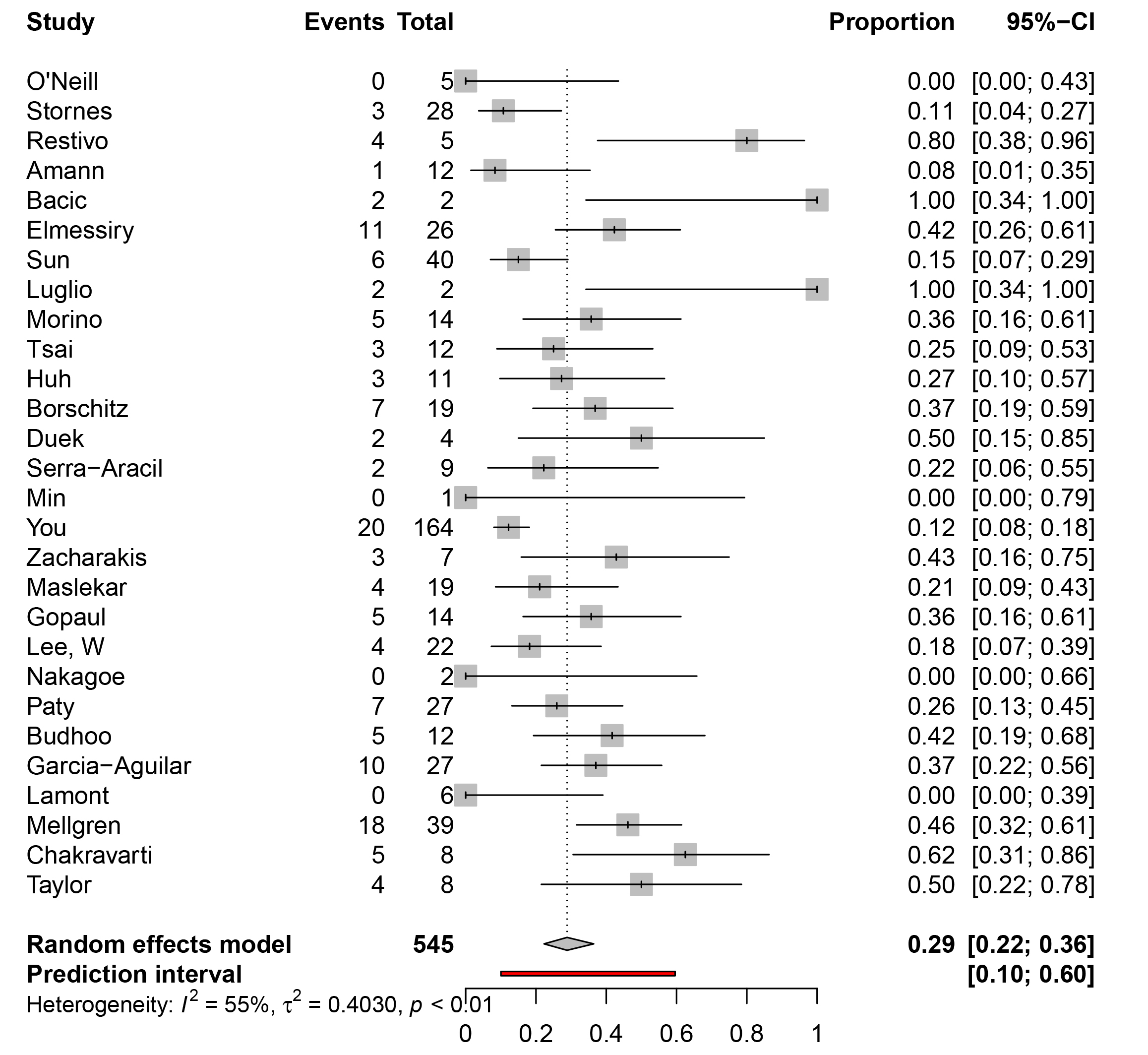


**Figure S2** **Forest plots of overall local recurrence of local excision followed by completion total mesorectal excision in patients with a) pT1 b) pT2 tumours. An inverse-variance random-effects model. Proportions with 95 per cent confidence intervals**

**a pT1**


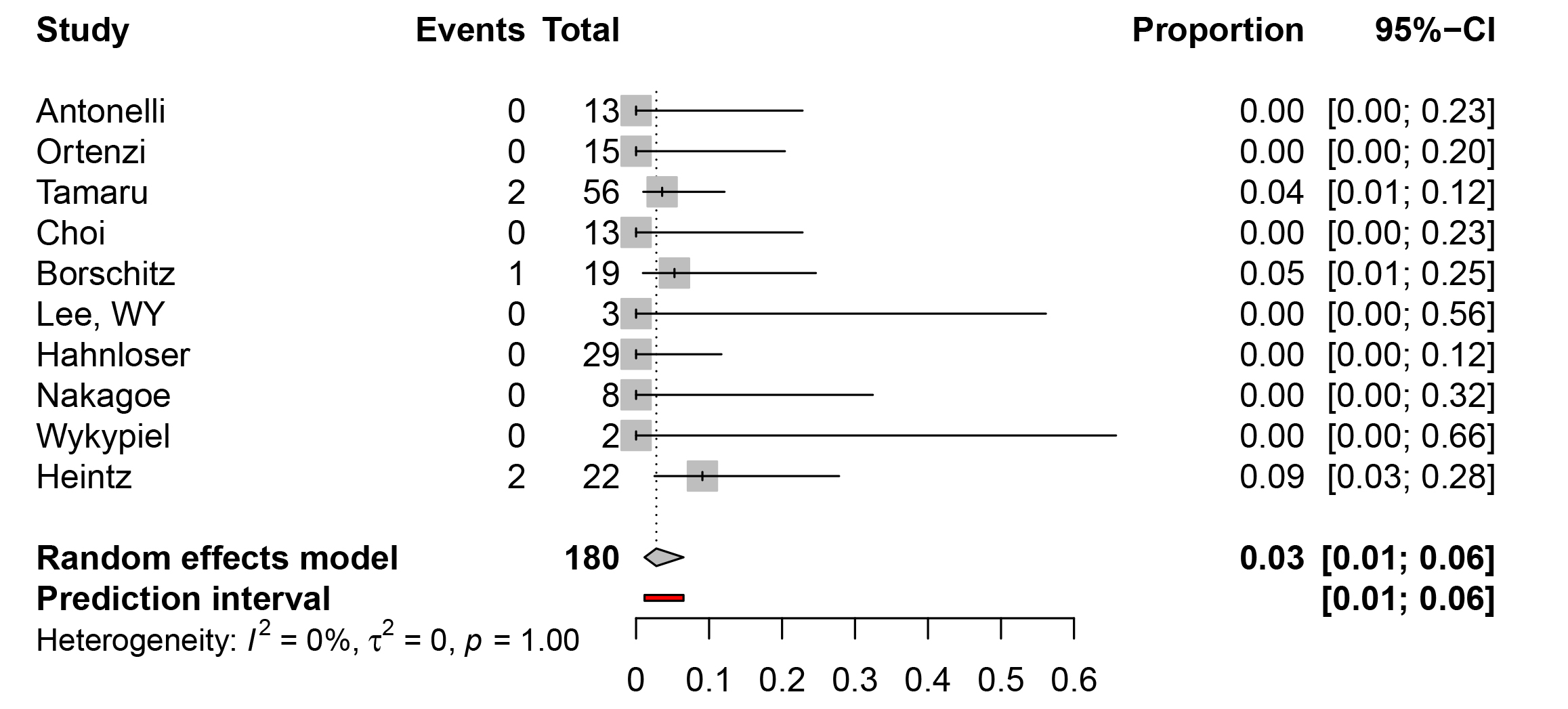


b pT2


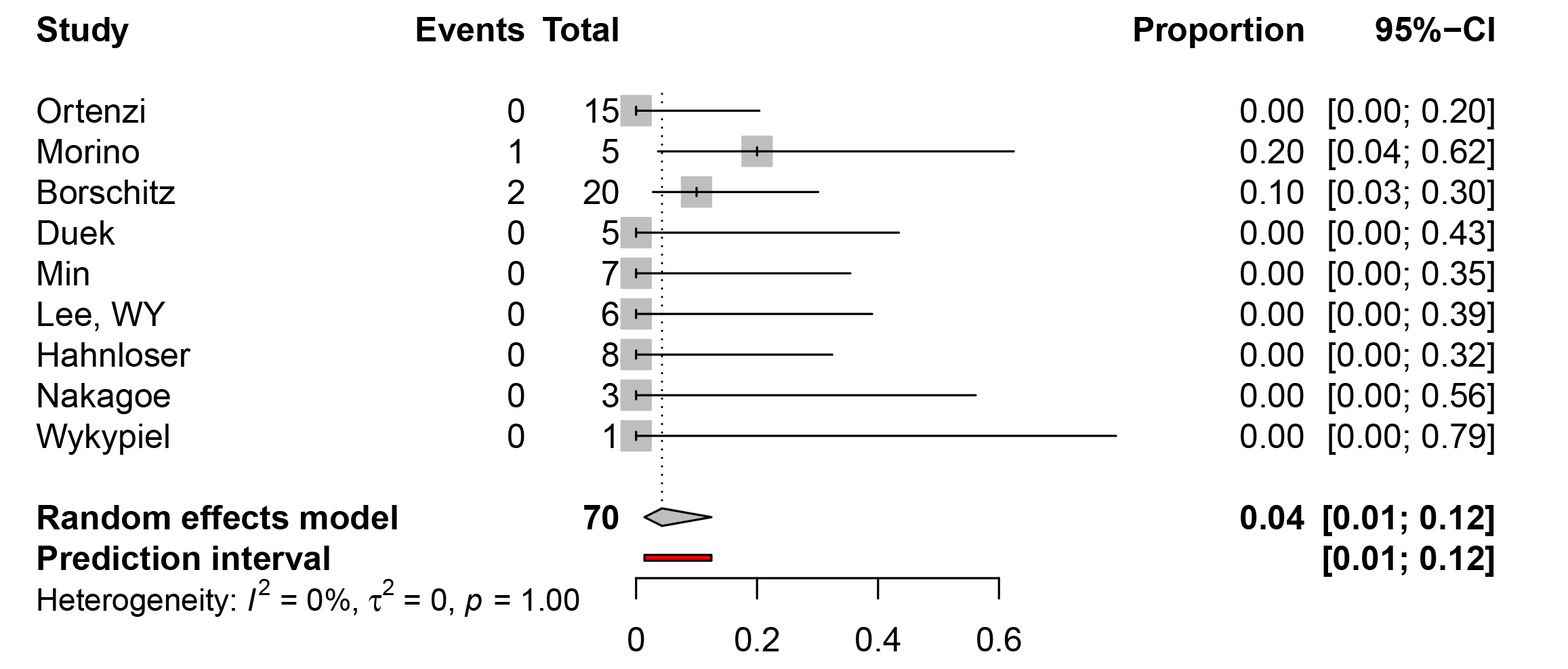


**Figure S3** **Forest plots of overall local recurrence of local excision followed by adjuvant (chemo)radiotherapy in patients with a) pT1 b) pT2 tumours. An inverse-variance random-effects model. Proportions with 95 per cent confidence intervals**

**a pT1**

**
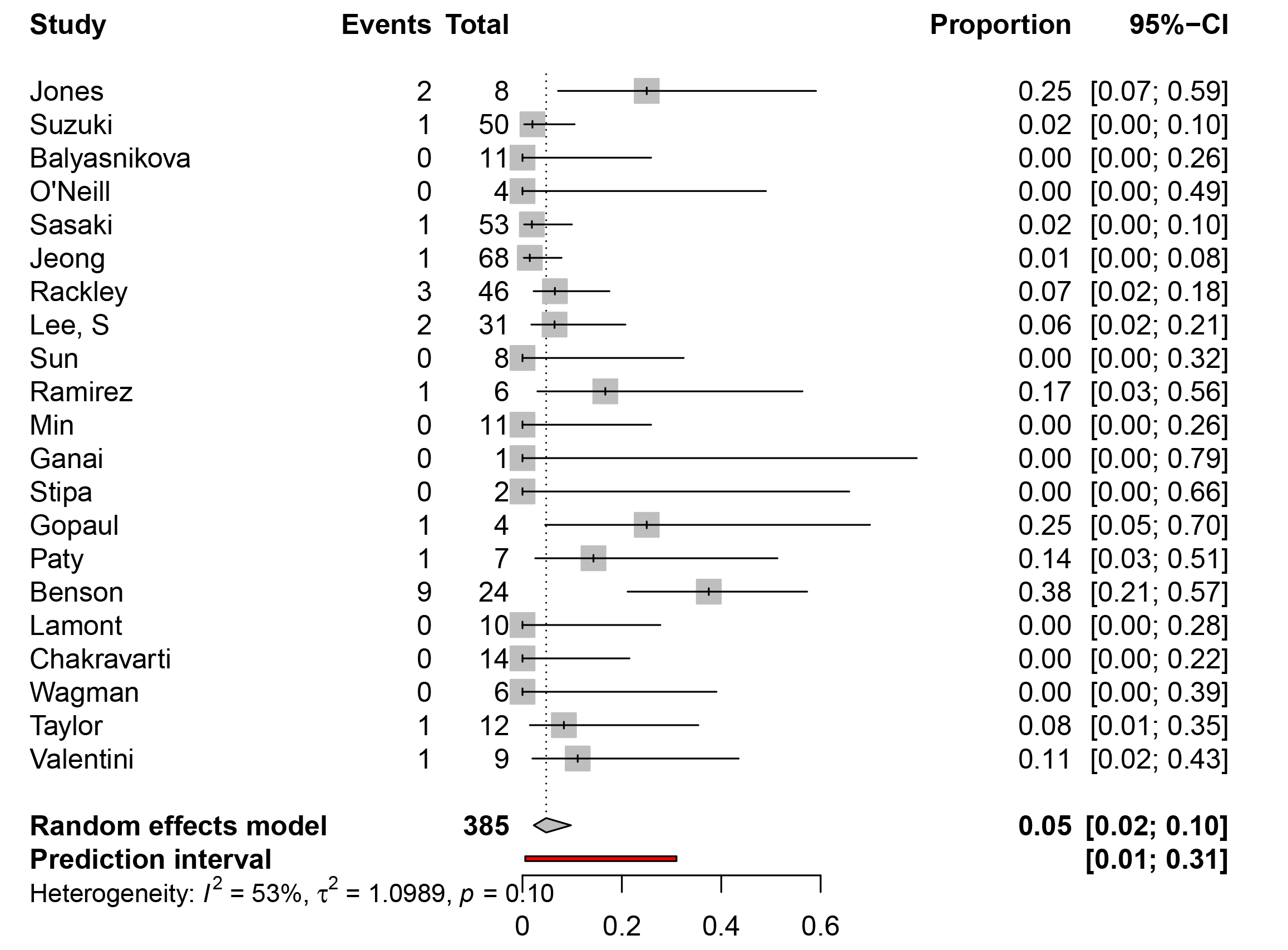
**

b pT2


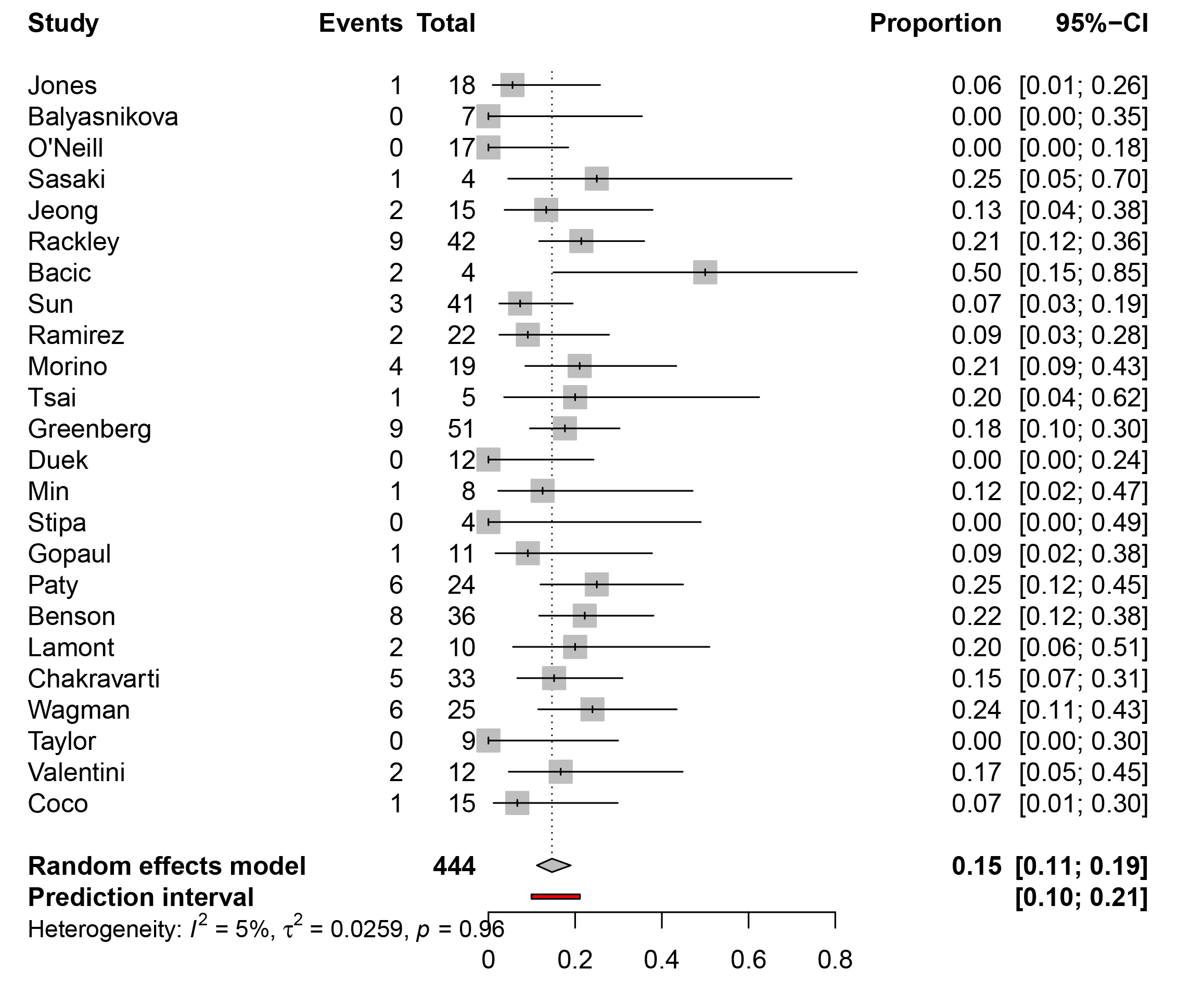


**Figure S4 Forest plots of overall local recurrence of local excision without additional treatment, subgroup analysis low- and high-risk T1 tumours a) low-risk pT1 and b) high-risk pT1 tumours. An inverse-variance random-effects model. Proportions with 95 per cent confidence intervals**

**a Low-risk pT1**

**
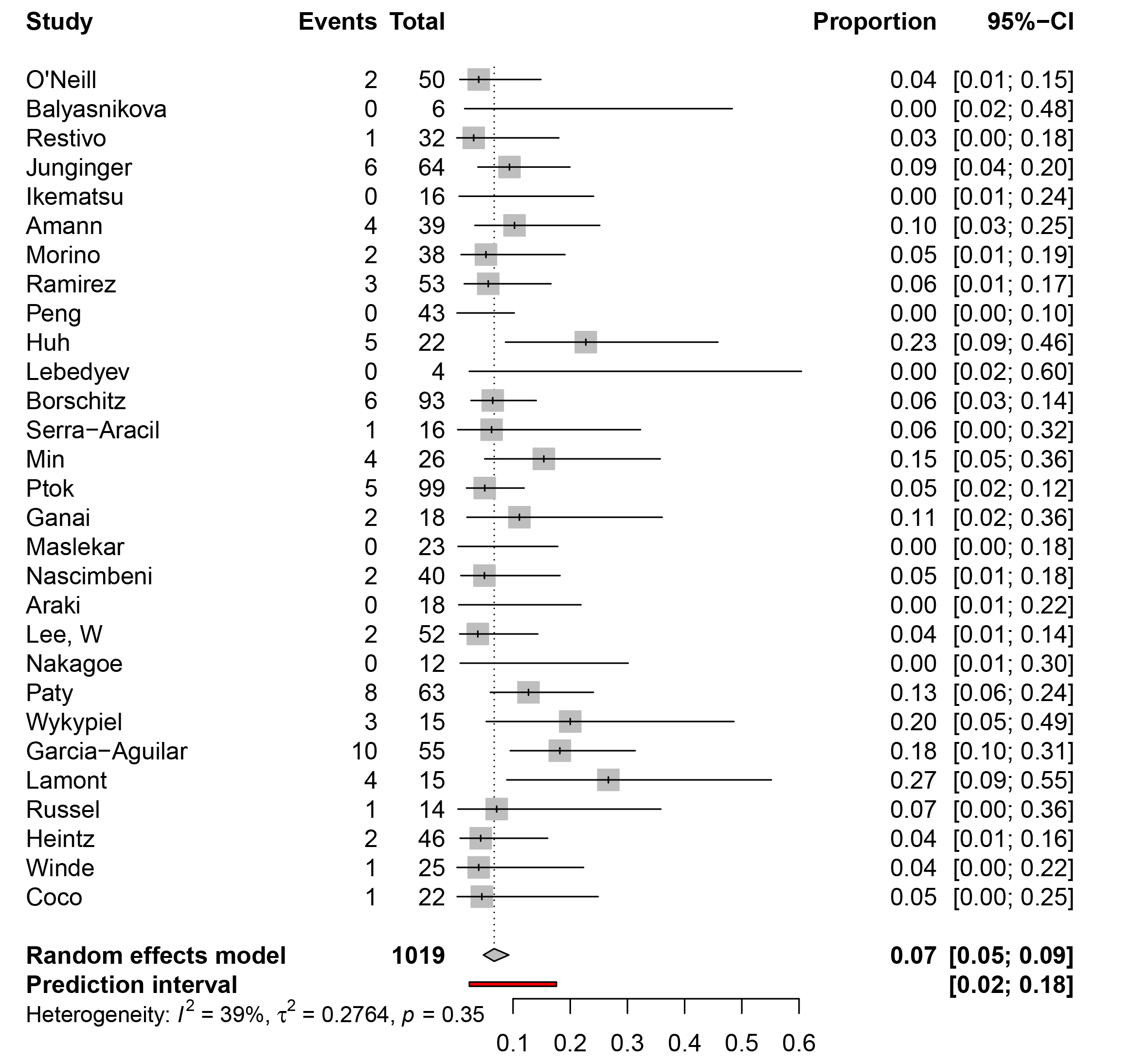
**

**b High-risk pT1**

**
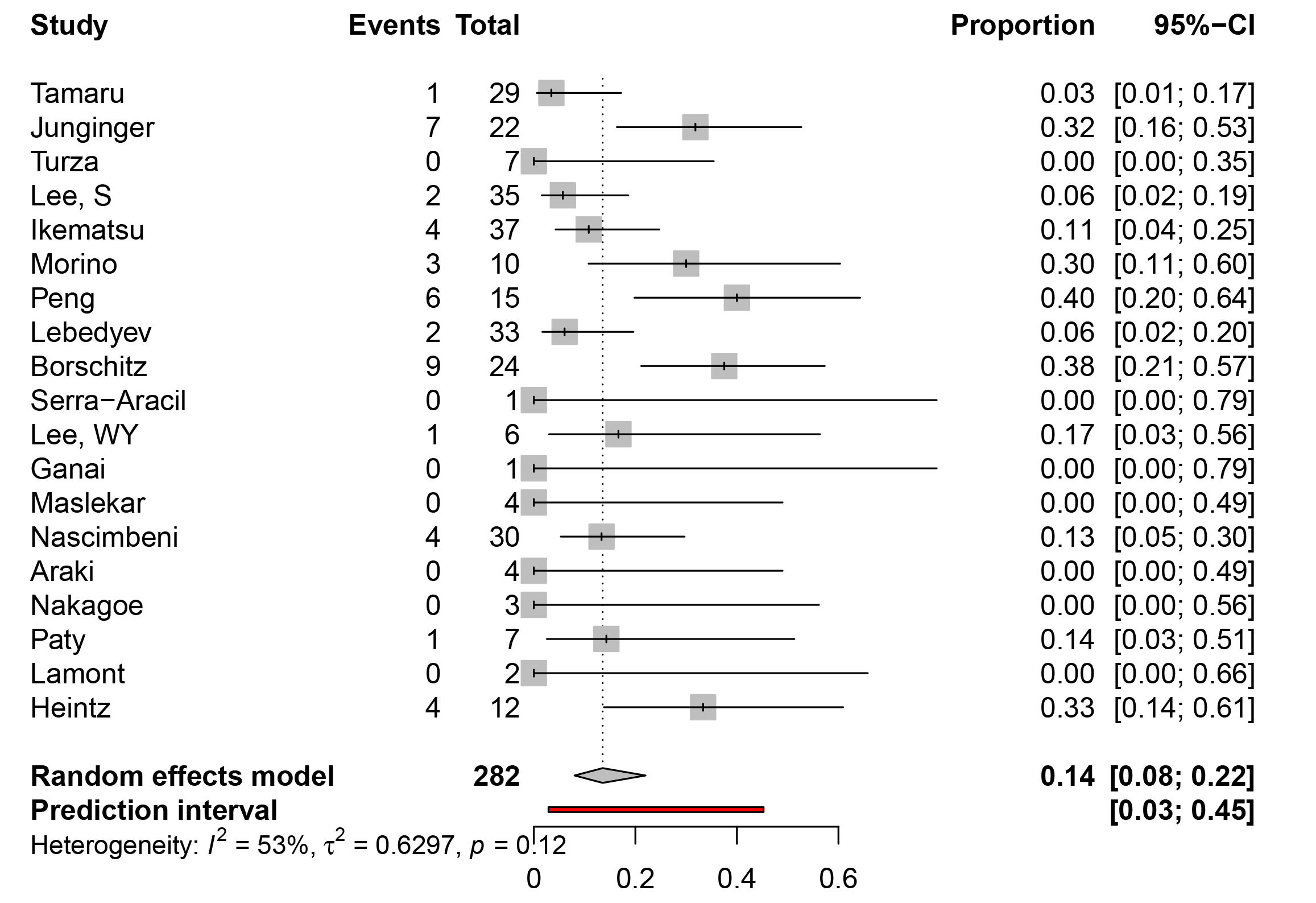
**

**Figure S5** **Forest plots of overall local recurrence of local excision followed by completion total mesorectal excision, subgroup analysis high-risk pT1 tumours. An inverse-variance random-effects model. Proportions with 95 per cent confidence intervals**

**a High-risk pT1**

**
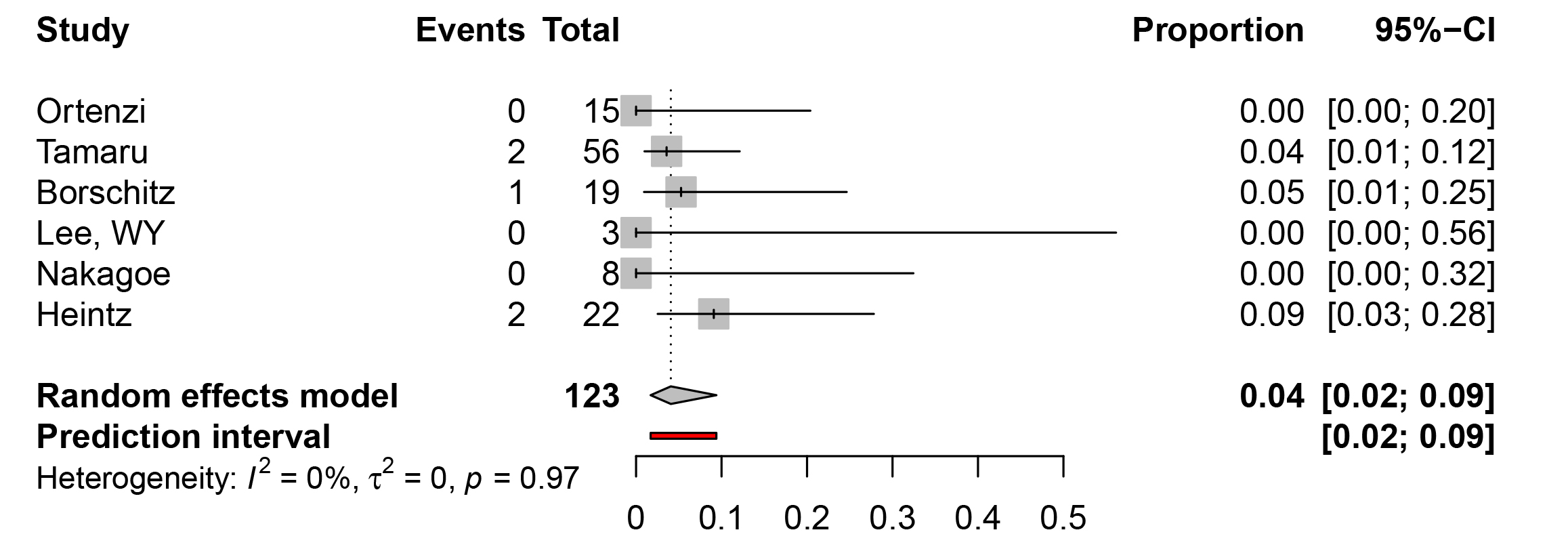
**

**Figure S6** **Forest plots of overall local recurrence of local excision followed by adjuvant (chemo)radiotherapy, subgroup analysis high-risk pT1 tumours. An inverse-variance random-effects model. Proportions with 95 per cent confidence intervals**

**a High risk pT1**

**
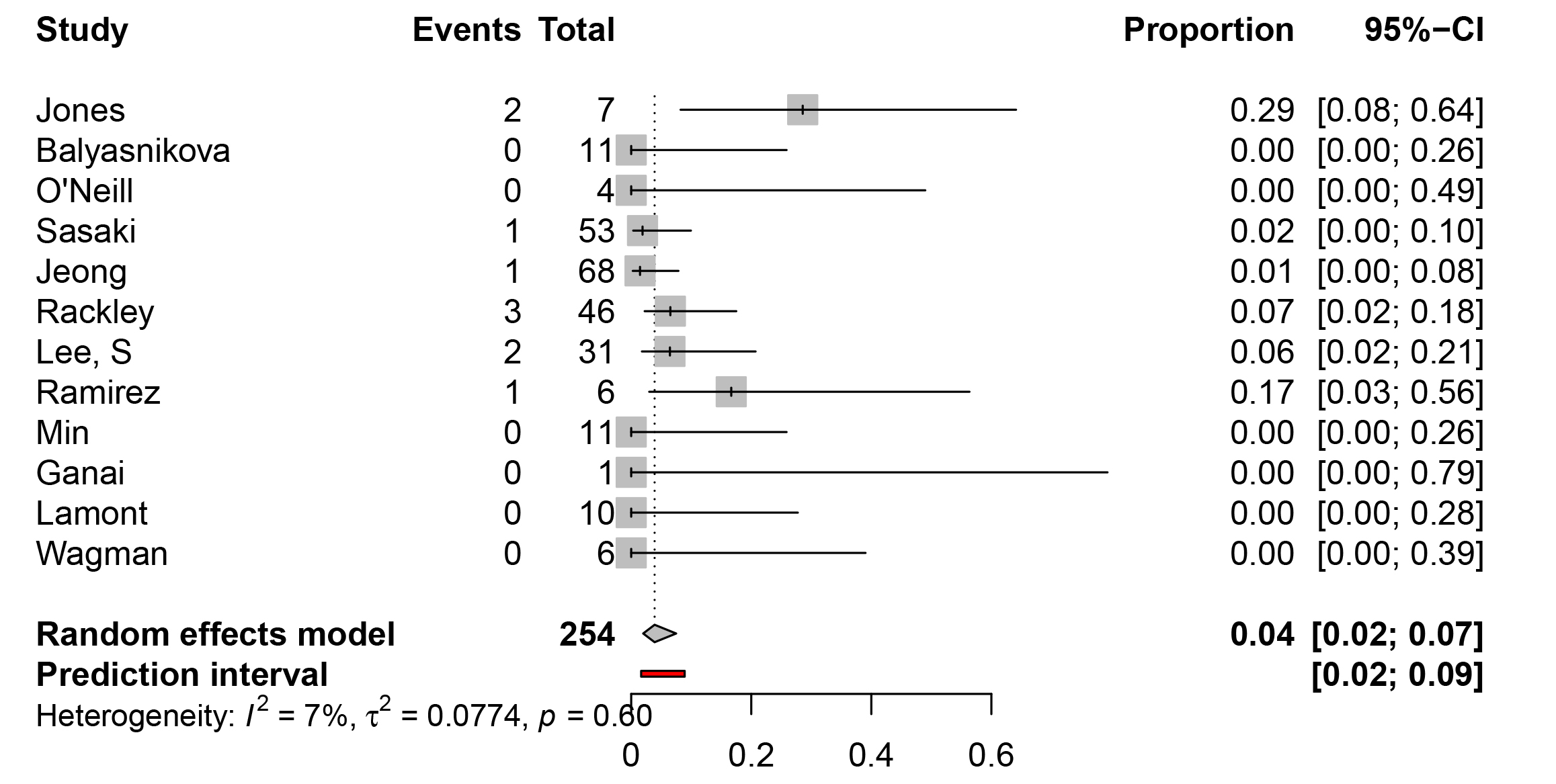
**

**Figure S7 Forest plots of overall distant recurrence of local excision without additional treatment in patients with a) pT1, b) pT2 tumours. An inverse-variance random-effects model. Proportions with 95 per cent confidence intervals.**

a pT1


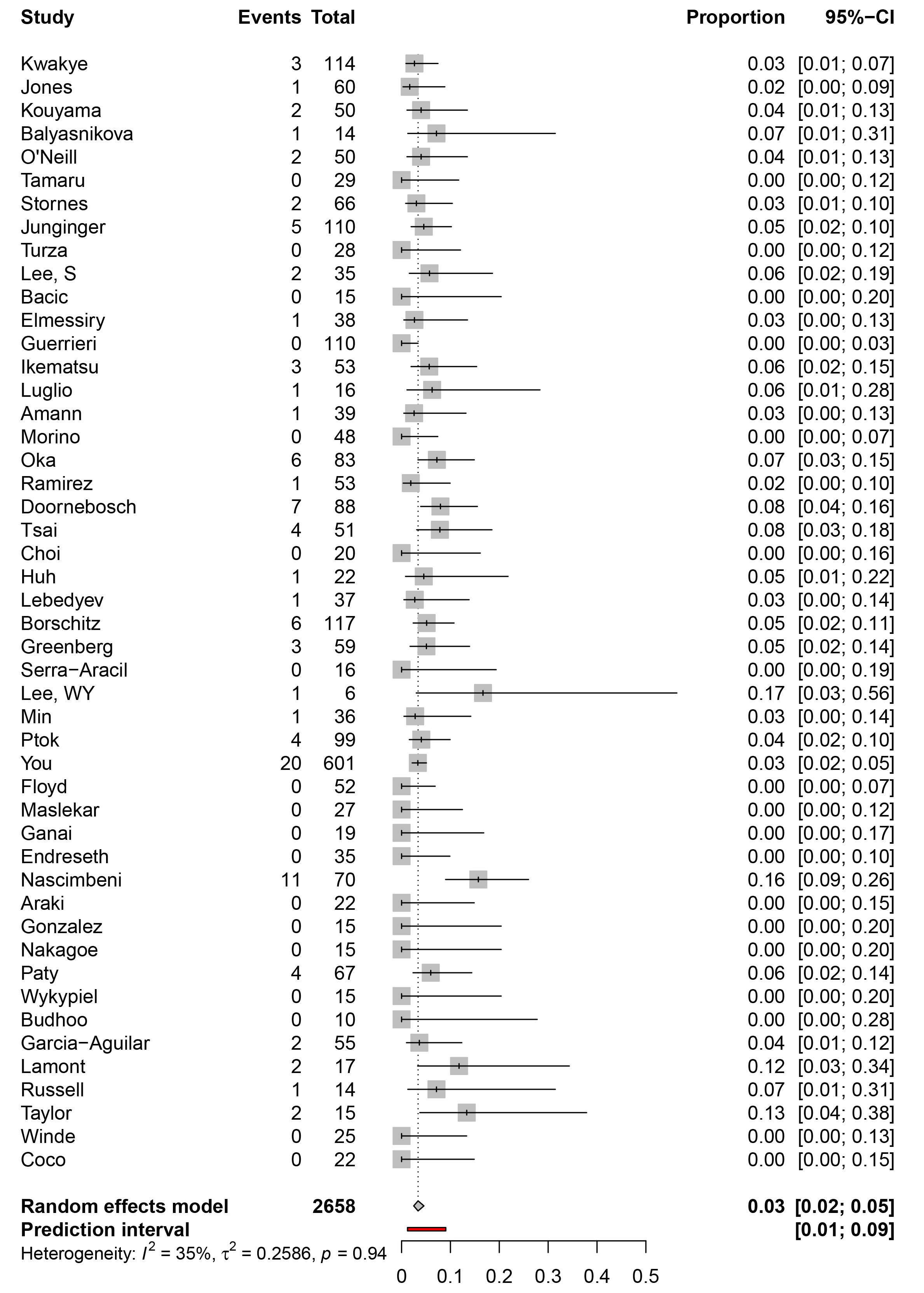


**b pT2**


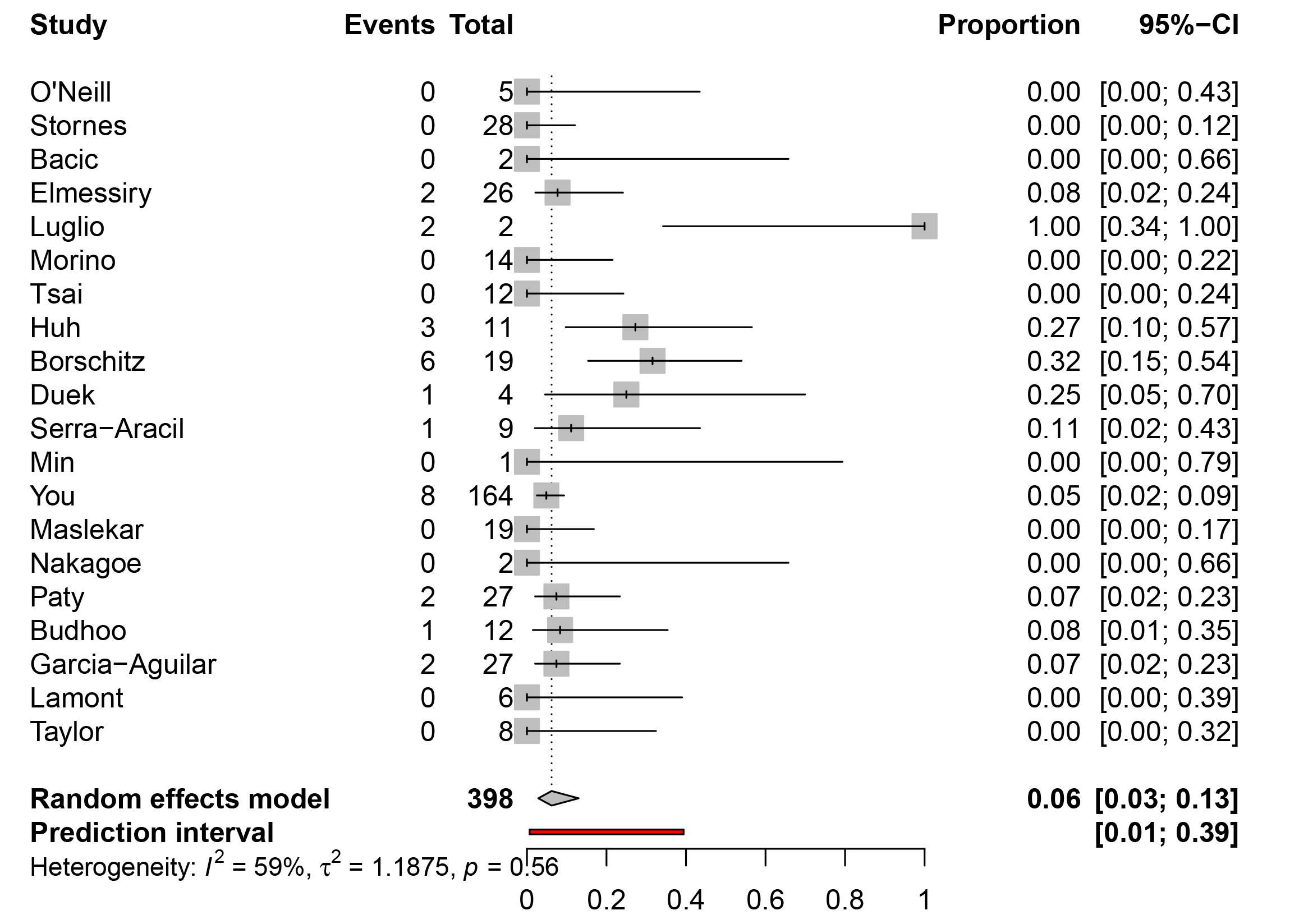


**Figure S8 Forest plots of overall distant recurrence of local excision followed by completion total mesorectal excision in patients with a) pT1 and b) pT2 tumours. An inverse-variance random-effects model. Proportions with 95 per cent confidence intervals**

a pT1


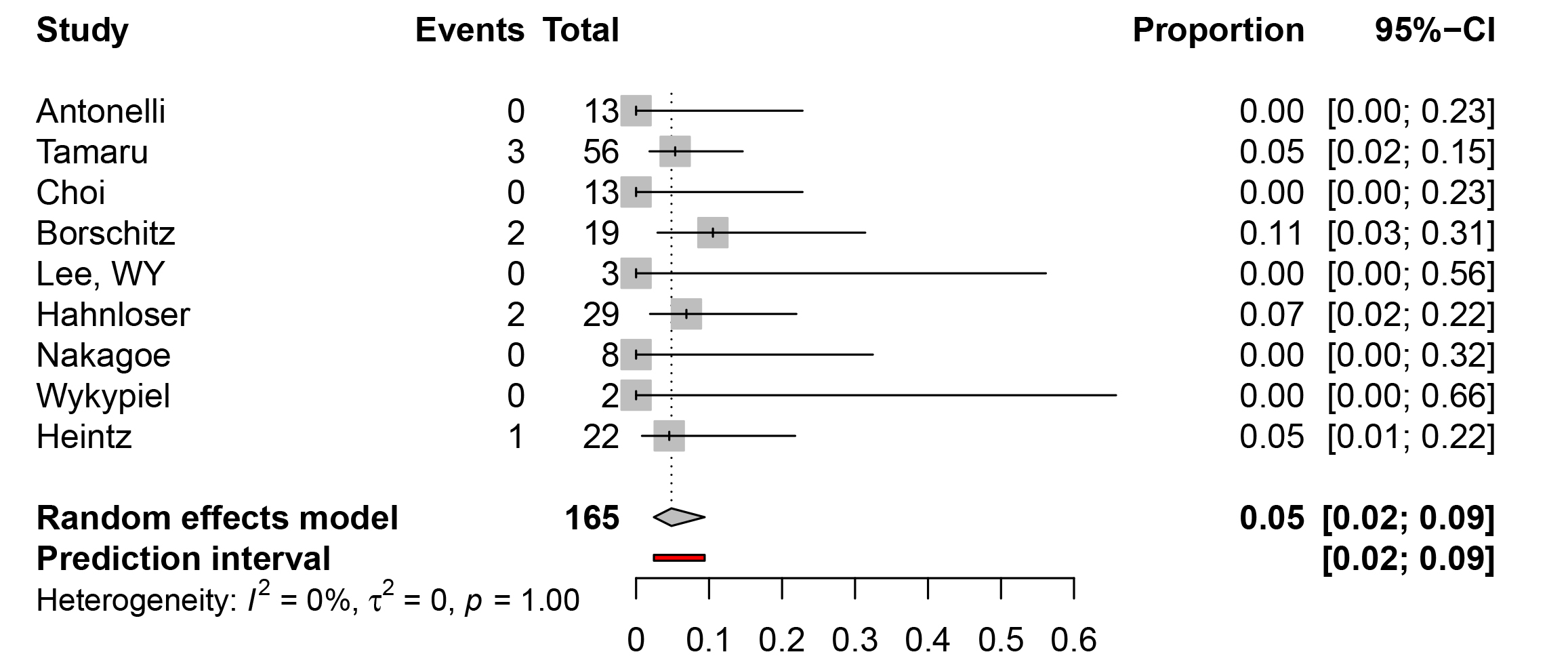


b pT2


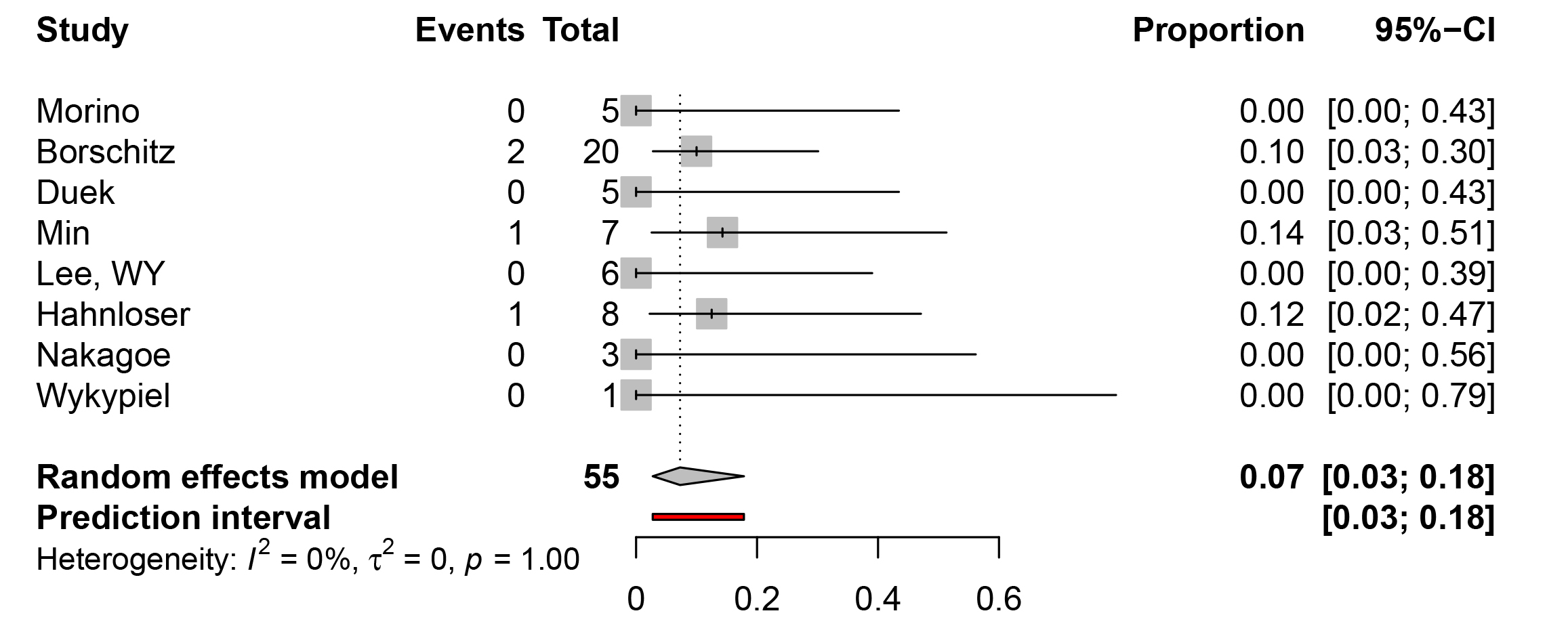


**Figure S9 Forest plots of overall distant recurrence of local excision followed by adjuvant (chemo)radiotherapy in patients with a) pT1 and b) pT2 tumours. An inverse-variance random-effects model. Proportions with 95 per cent confidence intervals**

a pT1


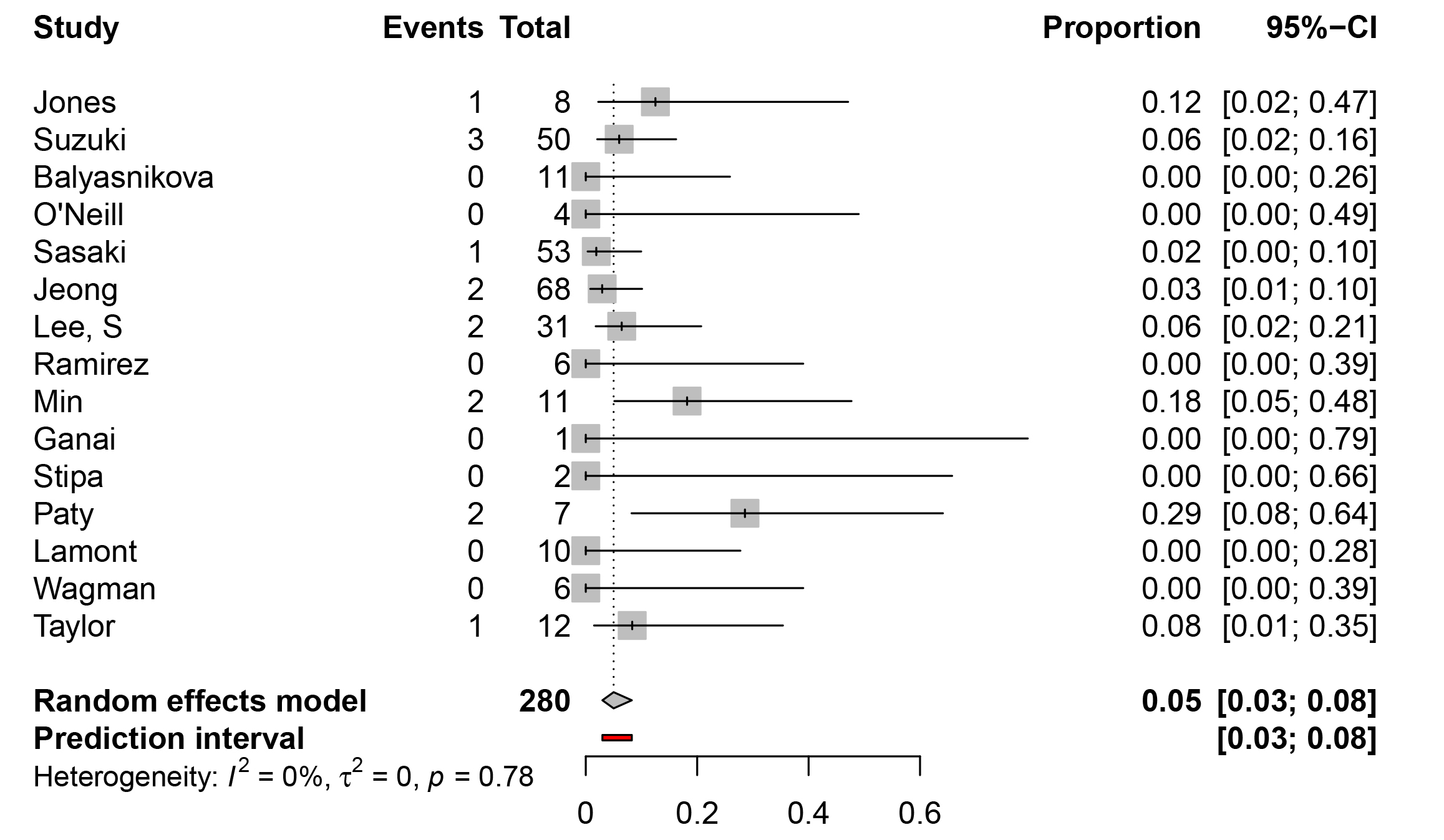


b pT2


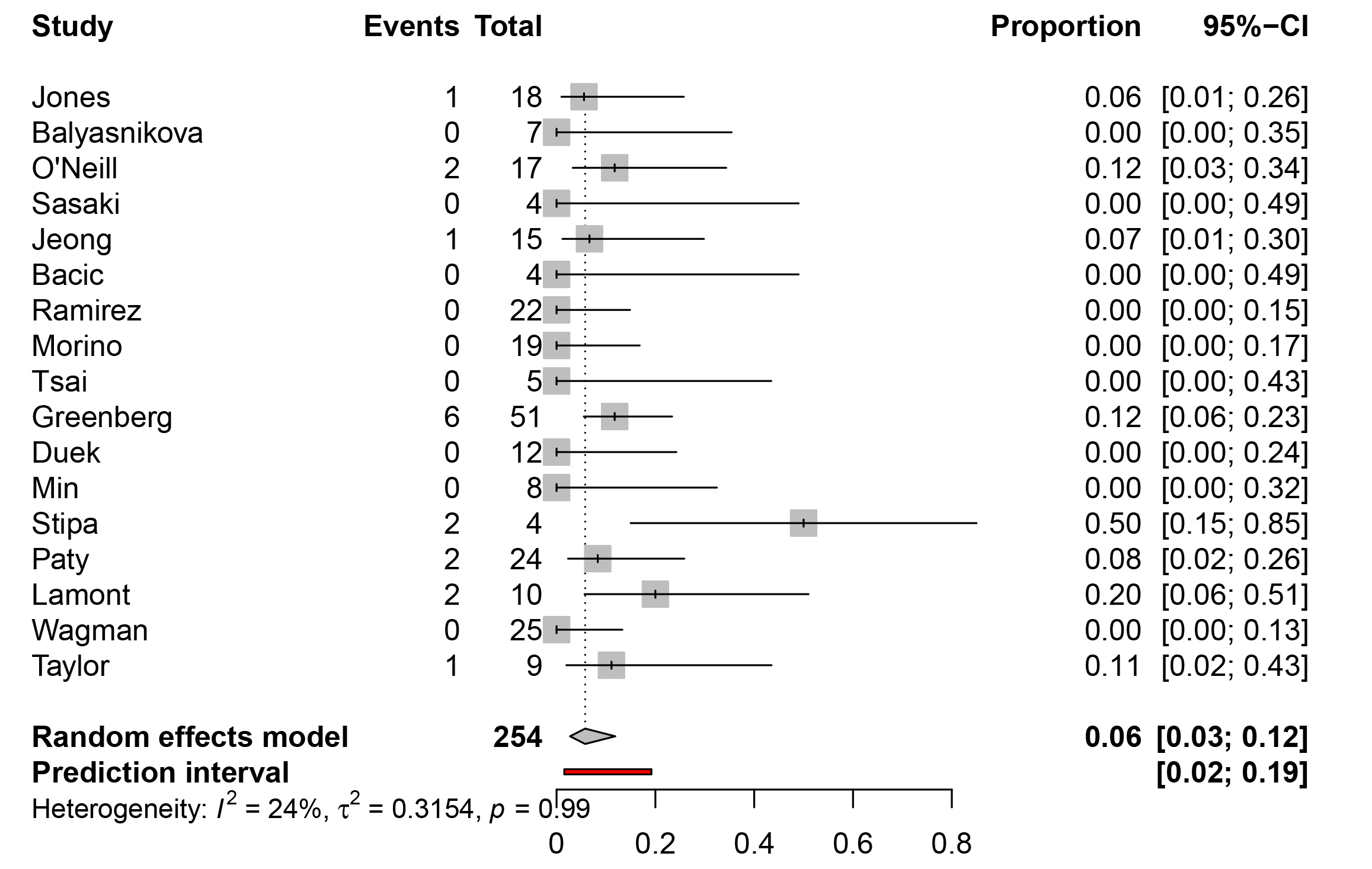

Supplement: Supplementary file 1 — Appendix S1 Search details of local excision without additional treatment Appendix S2 Search details of adjuvant (chemo)radiation and completion TME following local excision Table S1 Characteristics of studies on local excision without additional treatment for early rectal cancer Table S2 Characteristics of studies on local excision followed by completion TME for early rectal cancer Table S3 Characteristics of studies on local excision followed by adjuvant (chemo)radiation for early rectal cancer Table S4 Proportions of local recurrence, either local recurrence only or local recurrence and distant metastases. Table S5 Outcome data of local excision without additional treatment Table S6 Outcome data of local excision followed by completion total mesorectal excision Table S7 Outcome data of local excision followed by adjuvant (chemo)radiation Table S8 Outcome data of local excision without additional treatment, subgroup analysis low‐ and high‐risk pT1. Table S9 Outcome data of local excision followed by completion total mesorectal excision, subgroup analysis low‐ and high‐risk pT1 Table S10 Outcome data of local excision followed by adjuvant (chemo)radiotherapy, subgroup analysis low‐ and high‐risk pT1 Table S11 Quality assessment of studies on no additional treatment after local excision Table S12 Quality assessment of studies on completion total mesorectal excision and adjuvant (chemo)radiation following local excision Figure S1 Forest plots of overall local recurrence of local without additional treatment in patients with a) pT1 and b) pT2 tumours. An inverse‐variance random‐effects model. Proportions with 95 per cent confidence intervals Figure S2 Forest plots of overall local recurrence of local excision followed by completion total mesorectal excision in patients with a) pT1 b) pT2 tumours. An inverse‐variance random‐effects model. Proportions with 95 per cent confidence intervals Figure S3 Forest plots of overall local recurrence of local excision followed by a [file BJS-107-1719-s001.docx]
